# Long-term risk of chronic liver disease in patients with celiac disease: a nationwide population-based, sibling-controlled cohort study

**DOI:** 10.1016/j.lanepe.2024.101201

**Published:** 2025-01-09

**Authors:** Jialu Yao, Jiangwei Sun, Fahim Ebrahimi, David Bergman, Peter H.R. Green, Hannes Hagström, Benjamin Lebwohl, Daniel A. Leffler, Jonas F. Ludvigsson

**Affiliations:** a Department of Medical Epidemiology and Biostatistics, Karolinska Institutet, Stockholm, Sweden; b Department of Gastroenterology and Hepatology, University Digestive Health Care Center Basel – Clarunis, Basel, Switzerland; c Division of Digestive and Liver Diseases, Department of Medicine, Columbia University College of Physicians and Surgeons, New York, NY, USA; d Department of Medicine, Celiac Disease Center, College of Physicians and Surgeons, Columbia University Medical Center, New York, NY, USA; e Division of Hepatology, Department of Upper GI Diseases, Karolinska University Hospital, Stockholm, Sweden; f Department of Medicine, Huddinge, Karolinska Institutet, Stockholm, Sweden; g Department of Epidemiology, Mailman School of Public Health, Columbia University, New York, NY, USA; h Celiac Center, Beth Israel Deaconess Medical Center, Harvard Medical School, Boston, MA, USA; i Takeda Development Center Americas, Inc., Cambridge, MA, USA; j Department of Pediatrics, Örebro University Hospital, Örebro, Sweden

**Keywords:** Celiac disease, Chronic liver disease, Nationwide, Cohort

## Abstract

**Background:**

Celiac disease (CeD) may be associated with elevated liver enzymes. However, little is known about the risk of chronic liver disease (CLD) of various etiologies or major adverse liver outcomes (MALO) in CeD. We aimed to investigate the long-term risk of CLD in patients with CeD.

**Methods:**

Swedish nationwide cohort study. We identified 48,027 patients with biopsy-confirmed CeD between 1969 and 2017. Each patient was exactly matched with ≤5 general population reference individuals (n = 231,909) and followed through 2021. Flexible parametric survival models estimated adjusted hazard ratios (aHRs) of any and specific CLD (i.e., viral hepatitis, metabolic dysfunction-associated steatotic liver disease [MASLD], alcohol-related liver disease, and autoimmune liver disease) and MALO (compensated/decompensated cirrhosis, hepatocellular carcinoma, liver transplantation, and liver-related death).

**Findings:**

During a median follow-up of 16.0 years, 649 patients with CeD and 1571 reference individuals developed any CLD (incidence rate: 79.4 vs. 39.5/100,000 person-years). CeD patients had a higher risk of developing any CLD than reference individuals (aHR = 2.01, 95%CI:1.82−2.22). This risk remained elevated ≥25 years after diagnosis, giving one extra CLD case per 110 CeD patients until then. Positive associations were present for autoimmune liver disease (aHR = 4.86), MASLD (aHR = 2.54), and alcohol-related liver disease (aHR = 1.51). Individuals with CeD were at significantly higher risk of incident MALO (aHR = 1.54). Sibling comparisons and sensitivity analyses confirmed the main findings.

**Interpretation:**

CeD is associated with a persistently increased risk of any incident CLD, although the absolute risk is low. Physicians should be vigilant to early signs of liver dysfunction in patients with CeD.

**Funding:**

10.13039/100018353European Crohn's and Colitis Organisation, the 10.13039/501100003748Swedish Society for Medical Research (project#: PG-23-0315-H-02), 10.13039/501100006636FORTE (project#: 2016-00424), 10.13039/100007723Takeda, and the 10.13039/501100001711Swiss National Science Foundation (project#: P500PM_210866).

**Research in context**

**Evidence before this study**

We searched PubMed for evidence linking celiac disease (CeD) to chronic liver disease (CLD) and major adverse liver outcomes. Peer-reviewed articles in any language that were published from database inception through 27 September 2024 were considered. A search string for CeD was sequentially combined with those for CLD of various etiologies (including their former nomenclatures) and each component of major adverse liver outcomes. For example, following strings were used for the association between CeD and autoimmune liver disease: ((“celiac disease”) OR (“coeliac disease”) OR (CeD)) AND ((“autoimmune liver disease∗”) OR (“autoimmune hepat∗”) OR (AIH) OR (“primary biliary cholang∗”) OR (PBC) OR (“primary sclero∗ cholang∗”) OR (PSC)) AND ((“observational study”) OR (“longitudinal study”) OR (cohort) OR (case-control)). Positive associations of CeD with both steatotic and autoimmune liver diseases have been reported in meta-analyses and individual studies. However, most longitudinal studies are outdated and the only recent cohort focused on autoimmune liver disease. Therefore, there are still knowledge gaps in the risks for CLD of some specific etiologies (e.g., viral hepatitis, alcohol-related liver disease) and major adverse liver outcomes in patients with CeD.

**Added value of this study**

This nationwide cohort study provided up-to-date evidence on the CeD-associated risk of incident CLD. We observed a two-fold increased risk of developing any CLD, driven by autoimmune liver disease, metabolic dysfunction-associated steatotic liver disease, and alcohol-related liver disease, among patients with CeD than the general population. The risk was persistently elevated, leading to one extra CLD case per 110 patients during 25 years after CeD diagnosis. Subgroup analyses noted that CeD patients with a history of autoimmune or metabolic-related diseases were at further heightened risk for CLD.

**Implications of all the available evidence**

The increased risk for any CLD advocates clinical vigilance to signs of progressive liver diseases in patients with CeD to prevent major adverse liver outcomes. This aligns with current guidelines on liver enzyme monitoring in the medical follow-up of CeD patients. Our findings facilitate a more personalized approach by identifying high-risk populations for CLD. Moreover, the positive associations of CeD with both autoimmune and steatotic liver diseases call for research on the underlying immunological and metabolic mechanisms, as well as the hepatic impact of the gluten-free diet.

## Introduction

Celiac disease (CeD) is an immune-mediated disorder characterized by gluten-induced small intestinal villus atrophy (VA) and inflammation in genetically predisposed individuals.^1^ The disease imposes a substantial burden on both patients and society. The global prevalence of biopsy-confirmed CeD is about 0.7%,^1^ and almost one in every fifty individuals in Sweden will develop CeD throughout their life.^2^ Patients with CeD suffer from lifelong dietary restrictions and risks of complications both within and outside the small intestine.^3^

The liver is one of the organs at risk of CeD-associated complications. A recent meta-analysis estimated that about one-fifth of patients with CeD have elevated aminotransferase levels,^4^ which may signal underlying liver injury or imply a risk of developing chronic liver disease (CLD).^5^ Although more than 80% of CeD patients experience normalization of their liver enzyme levels following a gluten-free diet (GFD),^4^ evidence suggests that long-term dietary adherence is rather low.^6^ Metabolic alterations associated with CeD or a suboptimal adoption of the GFD that tends to be ultra-processed and have excess sugar may predispose to liver steatosis.^3^^,^^7^ In addition, various immunologic mechanisms link CeD to autoimmune liver disease including abnormal immune responses (e.g., elevated levels of pro-inflammatory cytokines and chemokines, aberrant T-lymphocyte homing), altered gut microbiome and associated metabolites,^8^, ^9^, ^10^ and shared risk genes (e.g., HLA-DQ2 genotypes for both CeD and autoimmune liver disease).^11^^,^^12^

Despite growing evidence for a positive association between CeD and CLD (summarized in Supplementary Tables S1a & S1b), most longitudinal studies are outdated with follow-ups ended fifteen to thirty years ago.^13^, ^14^, ^15^ Little is known about the risks for CLD of some specific etiologies (e.g., viral hepatitis and alcohol-related liver disease), or major adverse liver outcomes (MALO, a composite endpoint for compensated or decompensated cirrhosis, hepatocellular carcinoma (HCC), liver transplantation, and liver-related death). Moreover, the influence of persistent villus atrophy (VA) and familial risk factors (i.e., shared genetic and early environmental risk factors)^1^^,^^7^^,^^11^^,^^12^ on CLD risk remains unclear.

To address these knowledge gaps, we conducted a nationwide population-based cohort study to investigate the long-term risk of CLD in patients with biopsy-confirmed CeD. We hypothesized that patients with CeD have higher risks of any and specific CLD as well as a higher risk of developing MALO.

## Methods

### Data sources

This nationwide cohort was based on the Epidemiology Strengthened by histoPathology Reports in Sweden (ESPRESSO) cohort.^16^ ESPRESSO is a histopathology cohort which collects gastrointestinal (GI) biopsy reports from all 28 pathology departments in Sweden from 1965 and updated through 2017.^16^ Information in ESPRESSO includes the date of biopsy, anatomic location, and morphology (by the adopted Swedish version of the Systematized Nomenclature of Medicine [SNOMED] system).^16^ The Swedish National Patient Register (NPR) contains data on inpatient care since 1964 (nationwide since 1987) and specialized outpatient care since 2001.^17^ The Cause of Death Register contains all registered deaths in Sweden since 1961.^18^ The Swedish Cancer Register (established in 1958) covers >96% incident cancer cases in Sweden.^19^ The Cause of Death Register and the Cancer Register were used to complement the identification of HCC and liver-related death.

### Identification of CeD patients and comparison groups

We identified patients with CeD from the ESPRESSO cohort.^16^ Patients with CeD were eligible for the study if they were living in Sweden and had villus atrophy (VA, Marsh 3) in the small intestine other than the ileum (see Supplementary Table S2 for relevant codes for CeD) between 1969 and 2017. This algorithm had a positive predictive value (PPV) of 95% for biopsies between 1969 and 2008,^20^ and 99% for biopsies between 2009 and 2017.^21^ The index date for CeD patients (i.e., the date of CeD diagnosis) was the date of their first biopsy with VA.

There were two comparison groups. First, each patient with CeD was matched with up to five general population reference individuals from the Total Population Register (TPR),^22^ using exact matching by sex, birth year, calendar year of index date, and county of residence. Second, full siblings of CeD patients who had not been diagnosed with CeD at their sibling’s diagnosis date were identified and retrieved from the Swedish Multi-Generation Register, which is a component of the TPR.^23^ The index date for individuals in the comparison group was the date of them being matched.

We excluded any individual with a history of liver or alcohol-related conditions, human immunodeficiency virus infection, or liver transplantation identified from the NPR^17^ before the index date (see Supplementary Table S3 for the exclusion criteria).

### Identification of persistent VA and mucosal healing among CeD patients

A subset of CeD patients who had a follow-up biopsy within six months and five years after CeD diagnosis was identified. We divided them into the persistent VA group and the mucosal healing group by presence of VA (Marsh 3) in the follow-up biopsy (see Supplementary Table S2 for relevant codes). The index date for this subset of CeD patients was the date of their follow-up biopsy, and same exclusion criteria as in the general population comparison were applied.

### Follow-up and ascertainment of the outcomes

The primary outcome was any incident CLD. Secondary outcomes included specific CLD by etiology (i.e., viral hepatitis, metabolic dysfunction-associated steatotic liver disease (MASLD), alcohol-related liver disease, and autoimmune liver disease) and MALO (a composite endpoint for compensated or decompensated cirrhosis, HCC, liver transplantation, or liver-related death). In the main analysis, we also identified each type of autoimmune liver disease, which included autoimmune hepatitis (AIH), primary biliary cholangitis (PBC), and primary sclerosing cholangitis (PSC). When identifying HCC diagnoses, the Cause of Death Register and Cancer Register were combined with the NPR for better sensitivity (see Supplementary Table S4 for codes relevant to outcomes).^24^ The risk for each component of MALO was also reported. In addition, we presented the prevalences of any or specific CLD diagnosed before incident MALO while following patients with CeD.

For each outcome, we followed individuals from the index date until the first diagnosis of the outcome of interest, emigration, death, or the end of study (31 December 2021). Reference individuals and full siblings were censored when being diagnosed with CeD.

Liver transplantation and incident HCC were additional censoring events when following individuals for any or specific CLD. For diagnostic validity, we also censored individuals with incident alcohol or drug-related diagnoses, viral hepatitis, and autoimmune liver disease when following them for MASLD (same codes as for exclusion, see Supplementary Table S3), this approach yielded a PPV of 91% for MASLD.^25^^,^^26^

### Covariates

The following covariates were considered in addition to the matching variables. For categorical variables with missing values, a separate “missing” category was created. Country of birth (Nordics [Sweden, Denmark, Finland, Norway, and Iceland] or others) was collected from the TPR.^23^ Educational attainment (0–9 years, 10–12 years, ≥13 years, or “missing”), a proxy for socioeconomic status, was ascertained from the Swedish Longitudinal Integrated Database for Health Insurance and Labour Market Studies (LISA).^27^ The educational level data have been available since 1990, with a reported accuracy of 85% for individuals in LISA.^27^ For individuals whose age at the index date was <22 years, the highest educational level among their own or their parents’ was used. The number of healthcare visits, a proxy for regular healthcare-seeking behavior, was retrieved from the NPR and defined as the number of specialized outpatient visits or hospitalizations between two years and six months before the index date and categorized into four groups: 0, 1, 2–3, and ≥4. Finally, we considered the presence of autoimmune diseases before the index date identified from the NPR (see Supplementary Table S4 for related codes).

### Statistical analyses

We reported the absolute risk of each outcome with incidence rate (IR) and IR difference, and the relative risk with adjusted hazard ratio (HR), along with 95% confidence intervals (CIs). The flexible parametric survival model was used to allow the time-varying effect of CeD.^28^ Standardized cumulative incidence was also estimated using the same approach.^29^ The underlying time scale was time since the index date (CeD diagnosis in patients).

In the population-matched cohort, we conditioned our analysis on matching variables (sex, birth year, calendar year of index date, and county of residence) in model 1, and additionally on educational attainment, country of birth, number of healthcare visits, and history of autoimmune diseases in model 2. In the sibling comparison, we conditioned our analysis on covariates in model 2 plus a family identifier. In the comparison between patients with persistent VA and mucosal healing, the duration of CeD diagnosis was adjusted for in addition to covariates in model 2 used in the population-matched cohort.

### Subgroup and sensitivity analyses

We estimated the risks of any and specific CLD as well as MALO by sex (female/male), age at index date (<18, 18–<40, 40–<60, ≥60 years), calendar period at index date (1969–1989, 1990–2001, 2002–2009, and 2010–2017), country of birth (Nordics or others), educational attainment (0–9, 10–12, ≥13 years, or “missing”), number of healthcare visits between two years and six months before the index date (0, 1, 2–3, and ≥4), history of autoimmune diseases, and history of metabolic-related diseases (i.e., hypertension, diabetes mellitus, obesity, and dyslipidemia) (see Supplementary Table S4 for related codes).

Several sensitivity analyses were conducted. First, because histopathology-based CeD diagnosis became optional for pediatric cases in Sweden in 2012,^30^ childhood-onset (<18 years) patients who were included since then may have represented special cases (e.g., cases with borderline tissue transglutaminase 2 antibody value) that required biopsy for ascertainment. For the impact of changing diagnostic criteria, we ran a separate analysis in childhood-onset patients who were identified *before* 2012. Second, to account for missing data, we restricted the analyses to individuals who had data on educational attainment in LISA.^27^ Third, for potential surveillance bias and reverse causation, we estimated the risk of each outcome after discarding the first one and three years of follow-up in each study group. Fourth, because genetic predisposition is necessary for CeD onset, we regarded CeD as a lifetime exposure.^1^ The risk estimate was calculated with conditional logistic regressions while conditioning on all covariates in model 2 plus the temporal order (before/after) between the date of first CLD and CeD diagnosis.

Data analysis was conducted in Stata (version 16.1; StataCorp LP, College Station, TX), and R version 3.6.0. A two-sided *P* ≤ 0.05 was considered statistically significant.

### Role of the funding source

The funders had no role in the design and conduct of the study; collection, management, analysis, and interpretation of the data; preparation, review, or approval of the manuscript; and decision to submit the manuscript for publication.

## Results

Between 1969 and 2017, we identified 48,027 patients with biopsy-confirmed CeD (median age at diagnosis: 27.3 years; diagnosed <18 years: 40.7%; female: 62.7%) and 231,909 matched reference individuals from the general population (see Supplementary Fig. S1 for study population selection and Table 1 for baseline characteristics). Among patients with CeD, 66.5% had been diagnosed after 2002 and over one-third were followed for ≥20 years. Compared with reference individuals, patients with CeD tended to have more healthcare visits and a higher prevalence of metabolic-related or autoimmune diseases before the index date (Table 1).

**Table 1:** Characteristics of patients with CeD and their matched reference individuals, n (%).

	Patients with CeD	References
N	48,027	231,909
Age at index date, years		
Mean ± SD	31.6 ± 25.2	30.8 ± 25.0
Median (IQR)	27.3 (8.0–52.8)	25.9 (7.5–51.7)
<18	19,525 (40.7%)	97,289 (42.0%)
18–<40	10,438 (21.7%)	50,176 (21.6%)
40–<60	9337 (19.4%)	44,302 (19.1%)
≥60	8727 (18.2%)	40,142 (17.3%)
Female	30,115 (62.7%)	146,388 (63.1%)
Born in Nordic countries^a^		
Yes	46,068 (95.9%)	213,086 (91.9%)
No	1959 (4.1%)	18,818 (8.1%)
Missing	0	5 (0.0%)
Calendar period at index date		
1969–1989	4215 (8.8%)	20,704 (8.9%)
1990–2001	16,694 (34.8%)	81,140 (35.0%)
2002–2009	15,729 (32.8%)	75,763 (32.7%)
2010–2017	11,389 (23.7%)	54,302 (23.4%)
Educational attainment, years		
0–9	6888 (14.3%)	35,189 (15.2%)
10–12	19,403 (40.4%)	93,981 (40.5%)
≥13	18,773 (39.1%)	87,602 (37.8%)
Missing	2963 (6.2%)	15,137 (6.5%)
Number of healthcare visits^b^		
0	27,236 (56.7%)	160,578 (69.2%)
1	8057 (16.8%)	32,630 (14.1%)
2–3	6469 (13.5%)	22,381 (9.7%)
≥4	6265 (13.0%)	16,320 (7.0%)
History of metabolic-related diseases^c^	5719 (11.9%)	17,875 (7.7%)
Hypertension	3141 (6.5%)	13,029 (5.6%)
Diabetes	2593 (5.4%)	4641 (2.0%)
Obesity	290 (0.6%)	1707 (0.7%)
Dyslipidemia	1683 (3.5%)	6697 (2.9%)
History of autoimmune diseases^c^	5131 (10.7%)	6251 (2.7%)
Follow-up time, years		
Mean ± SD	17.0 ± 8.8	17.2 ± 8.7
Median (IQR)	16.0 (10.2–23.0)	16.1 (10.3–23.2)
0–0.9	771 (1.6%)	2640 (1.1%)
1–4.9	1863 (3.9%)	9370 (4.0%)
5–9.9	8949 (18.6%)	42,827 (18.5%)
10–19.9	19,969 (41.6%)	96,176 (41.5%)
≥20	16,475 (34.3%)	80,896 (34.9%)

CeD: celiac disease; IQR: interquartile range: SD: standard deviation.

a Nordic countries: Sweden, Denmark, Finland, Norway, and Iceland.

b Between two years and six months before the index date.

c Codes for metabolic-related diseases and autoimmune diseases are listed in Supplementary Table S4.

### Primary outcome: any incident CLD

**Fig. 1:**
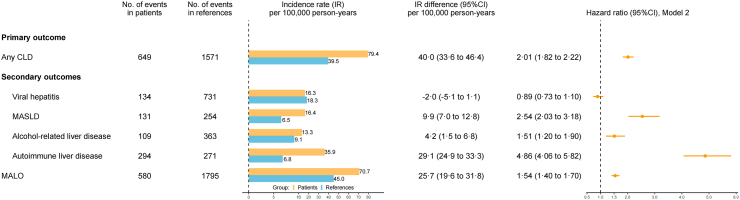
Incident chronic liver disease (CLD) and major adverse liver outcomes (MALO) in patients with celiac disease compared with their matched reference individuals. The hazard ratio was estimated with the flexible parametric survival model based on model 2. Adjusted covariates included matching variables (sex, birth year, calendar year of index date, and county of residence), educational attainment, country of birth, number of healthcare visits, and history of autoimmune diseases. CI: confidence interval; IR: incidence rate; MASLD: metabolic dysfunction-associated steatotic liver disease.

During a median follow-up of 16.0 years, there were 649 patients with CeD and 1571 reference individuals diagnosed with any CLD, giving an IR difference of 40.0 per 100,000 person-years (IR in CeD patients vs. reference individuals: 79.4 vs. 39.5, Fig. 1 & Supplementary Table S5).

**Fig. 2:**
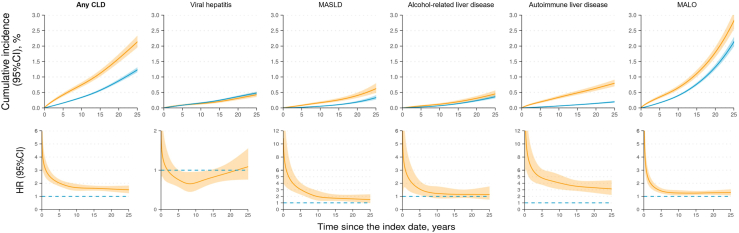
Standardized cumulative incidence and hazard ratio (HR) for chronic liver disease (CLD) and major adverse liver outcomes (MALO) in patients with celiac disease (yellow) compared with their matched reference individuals (blue), both with 95% confidence interval (CI). The standardized cumulative incidence and HR were estimated with the flexible parametric survival model based on model 2. Adjusted covariates included matching variables (sex, birth year, calendar year of index date, and county of residence), educational attainment, country of birth, number of healthcare visits, and history of autoimmune diseases. MASLD: metabolic dysfunction-associated steatotic liver disease.

After multivariable adjustment, patients with CeD had a two-fold elevated hazard of developing any CLD (aHR = 2.01 [95%CI: 1.82 to 2.22]) compared to reference individuals. The HR of any CLD was highest immediately after the diagnosis and remained elevated even after 25 years (Fig. 2). The differences in standardized cumulative incidence of any CLD, after 10 and 25 years of diagnosis, were 0.4% and 0.9%, respectively (Supplementary Table S6).

The subgroup analysis showed consistently elevated absolute and relative risks of developing future CLD among patients with CeD (Supplementary Table S7). There was a higher HR in females (aHR = 2.14 [1.88 to 2.43]) compared to males (aHR = 1.85 [1.59 to 2.17], *P*_*interaction*_<0.05), while IR differences were similar between the sexes (IR: 74.1 vs. 34.6 in females; 88.8 vs. 48.1 in males). In addition, both differences in IR and HRs of any CLD were further elevated in CeD patients who had autoimmune diseases (IR: 208.4 vs. 45.8, aHR = 4.30 [1.89 to 9.77]) or metabolic-related diseases (IR: 156.2 vs. 40.5, aHR = 3.81 [2.78 to 5.21]), with both *P*_*interaction*_<0.05 (Supplementary Table S7).

### Secondary outcomes: specific CLD

Compared with reference individuals, patients with CeD had higher absolute and relative risks of autoimmune liver disease (IR: 35.9 vs. 6.8 per 100,000 person-years; aHR = 4.86 [4.06 to 5.82], higher risks were observed for each of AIH, PBC, and PSC). In addition, patients with CeD were at higher risk of developing MASLD (IR: 16.4 vs. 6.5; aHR = 2.54 [2.03 to 3.18]), and alcohol-related liver disease (IR: 13.3 vs. 9.1; aHR = 1.51 [1.20 to 1.90]), but not viral hepatitis (IR: 16.3 vs. 18.3; aHR = 0.89 [0.73 to 1.10]) (Fig. 1 & Supplementary Table S5). The HR for autoimmune liver disease was persistently high during 25 years after diagnosis, whereas hazard elevations for MASLD and alcohol-related liver disease were mostly evident in the first five years (Fig. 2).

### Secondary outcome: MALO

During follow-up, there were 580 individuals with CeD developed MALO events vs. 1795 in reference individuals, yielding an aHR of 1.54 [1.40 to 1.70]. The most frequent event was compensated or decompensated cirrhosis (IR: 61.8 vs. 38.5 per 100,000 person-years; aHR = 1.60 [1.44 to 1.78]). CeD was also associated with increased hazards of developing HCC (aHR = 1.40 [1.03 to 1.91]), liver transplantation (aHR = 2.66 [1.48 to 4.79]), or dying of liver-related causes (aHR = 1.38 [1.11 to 1.72]). There were 23.8% of patients with CeD diagnosed with any CLD before incident MALO, among which autoimmune liver disease and alcohol-related liver disease were most frequently observed (Supplementary Table S8).

The risks for autoimmune liver disease, MASLD, alcohol-related liver disease, and MALO were increased in most subgroup strata (i.e., age, sex, place of birth, calendar period, educational attainment, and history of autoimmune or metabolic-related diseases), while the association between CeD and viral hepatitis was generally non-significant (Supplementary Table S7). The modifying effect of a history of metabolic-related diseases was seen for all secondary outcomes except viral hepatitis. There were higher IR differences and higher HRs of autoimmune liver disease, MASLD, alcohol-related liver disease, and MALO (all *P*_*interaction*_<0.05) in patients with any metabolic-related disease.

### Sensitivity analyses

Our findings were robust when restricting the analysis to childhood-onset patients who were diagnosed before 2012 (Supplementary Table S9, compared with results for childhood-onset patients in Supplementary Table S7), when restricting to individuals who had data on educational attainment, when excluding the first one or three years of follow-up, and when treating CeD as a lifetime exposure (Supplementary Table S10).

### Sibling comparison

We identified 31,176 patients with CeD who had at least one CeD-free sibling living in Sweden and compared them with 52,999 full siblings (Supplementary Table S11). Patients with CeD tended to be younger, were more commonly female, and had a higher prevalence of autoimmune diseases and metabolic-related diseases compared with their full siblings. Consistent with the findings from the general population comparison, patients with CeD had higher risks of developing any and specific CLD (except viral hepatitis) and MALO than their full siblings (Supplementary Table S12). The subgroup-specific risk patterns were similar to the general population comparison, and we observed a notable risk attenuation for autoimmune liver disease (Supplementary Table S13).

### Impact of persistent VA on risks of CLD and MALO

Among 9392 patients with CeD who had a follow-up biopsy performed within six months and five years after CeD diagnosis, there were 2768 patients with persistent VA and 6624 showed signs for mucosal healing (Supplementary Table S14). At the time of follow-up biopsy, patients with persistent VA were about 18 years older (in median), had a lower prevalence of females, and tended to have a lower educational level than patients with mucosal healing. Among investigated outcomes, only the hazard for liver-related death was significantly increased in patients with persistent VA, however the 95%CI was wide (aHR = 2.74 [1.04 to 7.20], Supplementary Table S15). Interestingly, patients with persistent VA had a lower hazard of incident MASLD, although the HR failed to reach statistical significance (aHR = 0.72 [0.31 to 1.64]).

## Discussion

In this Swedish nationwide cohort study, we observed a two-fold increased relative risk of developing any CLD among patients with CeD compared with the general population. The relative risk was highest immediately after the diagnosis, potentially due to reverse causation (i.e., elevated liver enzymes in undiagnosed CLD may have prompted case finding and detection of CeD, with underlying liver condition confirmed later)^5^^,^^31^ and detection bias (i.e., incidental findings during diagnostic work-ups for CeD). However, the risk remained persistently elevated, resulting in one extra CLD case per 110 CeD patients during the first 25 years. The absolute and relative risks for any CLD were further elevated in those who had any concomitant autoimmune or metabolic-related disease.

The CLD risk increase was primarily driven by a higher incidence of autoimmune liver disease, and to a lesser extent by MASLD and alcohol-related liver disease. As a consequence, individuals with CeD were at a 54% increased risk of developing MALO, including liver decompensation and liver-related death. However, neither of the risks for any CLD or MALO was lower in individuals achieving mucosal healing during follow-up. Sibling-controlled analyses suggested that the associations could not be explained by familial risk factors (i.e., shared genetics and early environmental risk factors).^1^^,^^7^^,^^11^^,^^12^

CeD may manifest with elevated liver enzymes,^3^^,^^5^^,^^31^^,^^32^ which is why testing for CeD is recommended when facing with abnormal liver chemistries.^5^^,^^31^ Although positive associations of CeD with various CLD have been consistently reported (Supplementary Table S1), most studies focused on prevalent liver conditions of only one or two etiologies. Our nationwide longitudinal study provides up-to-date evidence on the risk of incident CLD and is unprecedented in its wide range of investigated liver diseases. In addition, the long follow-up allows us to investigate MALO with sufficient power.

The associations of CeD with specific CLD as well as MALO largely confirm existing evidence in their directions of associations, despite in varied strengths given differences in study populations (e.g., data sources, baseline characteristics, study settings), definitions of conditions, study periods, and adjusted covariates.^13^^,^^15^^,^^33^^,^^34^ The pronounced risk elevation for autoimmune liver disease was also noted in a UK primary and secondary care cohort (from 2000 through June 2019), in which Conrad et al.^33^ observed a fivefold increased incidence for PBC/PSC in patients with CeD that fell within the range of ours (aHR point estimates: 3.1 for PBC and 6.3 for PSC). CeD-related risks of future MASLD, alcohol-related liver disease, and HCC have so far only been presented in a Swedish setting (i.e., ESPRESSO or inpatient register-based cohorts), and corresponding studies have reported risk estimates comparable to ours.^13^^,^^15^^,^^34^

A recent study by Schiepatti et al. reported higher risks of malignant complications and death among patients with persistent VA than those who achieved mucosal healing.^35^ In that study, persistent VA was associated with higher odds of poor GFD adherence, poor clinical response to the GFD, classical CeD presentation (i.e., malabsorption, diarrhea, weight loss, and growth failure),^36^ and older age at diagnosis.^35^ While their findings highlighted adverse clinical outcomes, in our study, the risk for any investigated CLD or MALO did not differ significantly by mucosal healing status. The null association could be attributed to the small sample size (i.e., 137 incident CLD in total), potential mucosal recovery after the first follow-up biopsy,^37^ and the possibility of unintentional gluten ingestion in the long term.^6^ Although not statistically significant, there was a notable tendency for a lower future risk of MASLD associated with persistent VA, which was consistent with our previous findings.^13^ Because obesity was less prevalent in the persistent VA group (Supplementary Table S14), we postulate that the MASLD risk conferred by a high-sugar, high-fat GFD may be offset by more severe malabsorptive symptoms due to poorer GFD adherence in patients with VA.^3^^,^^7^^,^^35^^,^^38^

While distinct pathogenic pathways exist among various CLDs, immune dysregulation and gut microbiome alteration are the most common denominators in the association between CeD and CLD. Patients with CeD are subject to intestinal barrier dysfunction and a higher risk of small intestinal bacterial overgrowth.^10^ Excessive exposure to food and bacterial antigens stimulates the intestinal immune system, resulting in increased levels of pro-inflammatory cytokines (e.g., interferon-γ, interleukin-15) and chemokines (e.g., CC-chemokine ligand 20).^8^ These chemicals, entering the liver through portal circulation, eventually induce local inflammation and loss of self-tolerance via T-cell-mediated immune responses.^9^ In addition, subsequent to gut microbial perturbations, altered microbial metabolites in patients with CeD also contribute to the liver damage and steatosis via bile acid signalling pathways (mediated by e.g., the farnesoid X receptor).^9^^,^^10^

Some CLDs may be linked with CeD in more specific ways. Liver diseases of autoimmune etiology share risk genes with CeD at the HLA region,^11^^,^^12^ and their impact is implied in the large risk attenuation when comparing patients with CeD with their full siblings (Supplementary Table S12). The development of steatotic liver diseases can be driven by a GFD with potentially unbalanced nutritional content.^3^^,^^7^ Such a GFD induces fast weight gain especially in underweight patients.^39^ In turn, this predisposes to MASLD through worsened metabolic profiles and accelerated liver steatosis.^38^ On top of liver steatosis, the increased risk for alcohol-related liver disease can be explained by a higher prevalence of alcoholism in patients with CeD,^40^ potential detection and surveillance biases, and an increased susceptibility (i.e., a higher blood alcohol level due to a lower body mass and a greater gut permeability to bacterial antigens)^8^^,^^39^ to the hepatotoxic effect of alcohol.

This nationwide cohort study is, to date, the largest longitudinal study to investigate the association between CeD and CLD. We had a virtually complete follow-up of included individuals, thanks to the universal healthcare and the population-based registers in Sweden. The included patients with CeD were well characterized with the histopathology data and a pre-validated algorithm (i.e., PPV≥95%).^20^^,^^21^ Multiple linkages across the registers enabled data collection for covariate adjustment and subgroup analyses. These merits of our data minimized the selection and information biases and facilitated risk calculations with better precision. Moreover, the potential roles of shared genetics and early environmental risk factors were additionally accounted for via the comparison with CeD-free full siblings.^1^^,^^7^^,^^11^

This study also has limitations. First, despite the growing importance of serology in diagnosing CeD, our study only included patients identified through histopathology. To account for evolving diagnostic paradigms which mainly affected children in the Swedish context,^2^ we ran a sensitivity analysis in childhood-onset patients diagnosed before 2012 (i.e., before the introduction of serology-based diagnosis) and observed similar associations. Second, due to lack of primary care data and incomplete coverage of specialist care in the NPR,^17^ some milder cases of CLD were not identified. Moreover, the incidence of typically asymptomatic liver diseases, particularly MASLD, may have been underestimated. Presumably, missing outcome data occurred more frequently in CeD patients who were inherently more susceptible to elevated liver enzymes,^4^ therefore diluting the association towards null. However, patients with CeD were also more likely to have underlying liver conditions discovered due to diagnostic work-ups and medical follow-ups, leading to detection and surveillance biases that could inflate the risk estimates. Although we could not rule out their influence, the persistent risk elevation (Fig. 1) and robust results in sensitivity analyses (Supplementary Table S10) suggested that they are unlikely to fully explain observed associations. Third, while PPVs for some liver diagnoses were high (e.g., 91% for MASLD^25^^,^^26^ and 91% for cirrhosis^41^), there are knowledge gaps in the validity for viral hepatitis, alcohol-related liver disease, and autoimmune liver disease. To ensure consistency and validity in outcome identification, we applied codes in peer-reviewed literature.^42^^,^^43^ In addition, the PPVs for most chronic conditions in the NPR were generally high (85–95%).^17^ Fourth, while the PPV of identifying MASLD through non-alcoholic fatty liver disease is high,^25^^,^^26^ we could not discern malnutrition-associated steatotic liver disease from MASLD due to the absence of specific diagnostic codes and nutritional status data. Fifth, we did not have negative predictive values for the algorithms that identify CeD and liver diseases.

Sixth, some granular data were not available. In the absence of data on laboratory tests (e.g., liver function tests, T-cell clonality analyses), we were not able to tell whether an incident CeD may have been detected in case finding following elevated liver enzymes, and to discern nonresponsive or refractory cases.^3^^,^^31^ We also lacked dietary data to investigate the impact of the GFD on CLD. Seventh, even though alcohol was an established lifestyle risk factor for CLD,^7^ we acknowledge potential under-reporting of alcohol use in the NPR. Eighth, we urge caution when extrapolating our findings from Sweden to other settings, given the geographical and ethnical variation in HLA genotypes^1^^,^^7^^,^^11^^,^^12^ and environmental risk factors (e.g., gluten intake, alcohol use)^1^^,^^7^ predisposing to CeD or CLD. Another challenge for generalizability is the cross-country difference in clinical paradigms (e.g., different follow-up routines for patients with CeD).^1^ Finally, given the observational nature of this study, we do not claim any causal relationship between CeD and CLD.

The long-lasting association between CeD and any CLD in our study supports liver enzyme monitoring in the medical follow-up of CeD, which has been widely recommended in current guidelines.^3^^,^^32^ Clinicians should be vigilant for autoimmune liver disease or steatotic liver disease due to metabolic dysfunction or alcohol consumption to avoid subsequent MALO, especially during the first five years after CeD diagnosis. Notably, the risk for any CLD was not significantly reduced in patients who achieved mucosal healing than those with persistent VA.

Considering the low absolute risk (i.e., only one additional case of CLD expected among every 110 patients within 25 years after CeD diagnosis), a cost-effective and patient-centred strategy includes: informing CeD patients about established CLD risk factors (e.g., alcohol consumption and a nutritionally-unbalanced diet)^7^; being attentive to metabolic parameters (e.g., body mass index, fasting serum glucose level) when patients initiate a GFD; and evaluating and monitoring the GFD of those exhibiting elevated aminotransferase levels. This strategy applies even to patients who respond well to the GFD (e.g., achieve mucosal healing in the follow-up). In addition, given the high subgroup risks, patients with comorbid autoimmune or metabolic-related diseases may need more frequent follow-ups regarding diet and liver enzyme levels. Moreover, the high risk for autoimmune liver disease advocates special attention to CeD patients with genetic predispositions to this condition (e.g., genetic risk alleles at HLA-DRB1),^12^ and screening for related autoantibodies might be considered in case of cryptogenic hypertransaminasemia.^5^

In conclusion, CeD is associated with a two-fold increased relative risk of any CLD (including autoimmune liver disease, MASLD, and alcohol-related liver disease) for ≥25 years after diagnosis. High-risk populations for any CLD included individuals with a history of autoimmune or metabolic-related diseases. Clinicians should be vigilant to signs of progressive liver disease such as elevated liver enzymes in patients with CeD to prevent the long-term risk of developing MALO.

## Contributors

Study concept and design: JY, JS, JFL, FE, DB, PHRG, HH, BL, and DAL.

Acquisition of data: JFL.

Drafting of the manuscript: JY and JFL.

Interpretation of data, and critical revision of the manuscript for important intellectual content: JY, JS, FE, DB, PHRG, HH, BL, DAL, and JFL.

Statistical analysis: JY.

Funding acquisition: JFL.

Administrative, technical, or material support: JFL.

Guarantors: JY and JFL have directly accessed and verified the underlying data reported in the manuscript and take responsibility for the integrity of the data, the accuracy of the data analysis, and the decision to submit the manuscript.

## Data sharing statement

The data set cannot be shared directly under current legislation for data protection and must be requested directly from the respective registry holders, Statistics Sweden (information@scb.se) and the Swedish National Board of Health and Welfare (registerservice@socialstyrelsen.se), after approval by the Swedish Ethical Review Authority.

## Declaration of interests

All authors have completed the ICMJE uniform disclosure form and declare: FE has served as an advisory board member for Boehringer Ingelheim. HH’s institutions have received research funding from Astra Zeneca, EchoSens, Gilead, Intercept, MSD, Novo Nordisk and Pfizer. HH has served as a consultant or been on advisory boards for Astra Zeneca, Bristol Myers-Squibb, MSD, Novo Nordisk, and Takeda, and has been or is part of hepatic events adjudication committees for Arrowhead, Boehringer Ingelheim, GW Pharma, and KOWA outside the submitted work. DAL receives a salary as an employee of Takeda Pharmaceuticals. JFL has coordinated a study on behalf of the Swedish IBD quality register (SWIBREG). That study received funding from Janssen Corporation. JFL has also received financial support from MSD developing a paper reviewing national healthcare registers in China. JFL also has a research collaboration on celiac disease with Takeda. The other authors report no disclosures relevant to the manuscript.
