# Supplementary material for: Long-term risk of chronic liver disease in patients with celiac disease: a nationwide population-based, sibling-controlled cohort study
Source: Lancet Reg Health Eur. 2025 Jan 9;50:101201. doi: 10.1016/j.lanepe.2024.101201 (PMC11910788; doi:10.1016/j.lanepe.2024.101201)
Supplement: Supplementary Figure S1 and Tables S1–S15 [file mmc1.doc]

**Supplementary material**

**Long-term risk of chronic liver disease in patients with CeD: a nationwide population-based, sibling-controlled cohort study**

**Yao et al.**

**Table of Contents**

[Figure S1 Flow chart for study population selection 2](#__RefHeading___Toc178244678)

[Table S1a Synthesized or population-based evidence on the CLD risk in CeD 3](#__RefHeading___Toc178244679)

[Table S1b Synthesized or population-based evidence on the CeD risk in CLD 10](#__RefHeading___Toc178244680)

[Table S2 SNOMED codes defining CeD ^a^ 12](#__RefHeading___Toc178244681)

[Table S3 Definitions of exclusion criteria 13](#__RefHeading___Toc178244682)

[Table S4 Definitions of outcomes, comorbidities, and medications 14](#__RefHeading___Toc178244683)

[Table S5 Incident CLD and MALO in patients with CeD and their matched reference individuals 17](#__RefHeading___Toc178244684)

[Table S6 Cumulative incidence difference (95%CI) of incident CLD and MALO during follow-up in individuals with CeD, compared with their matched reference individuals 18](#__RefHeading___Toc178244685)

[Table S7 Subgroup analyses of incident CLD and MALO in patients with CeD and their matched reference individuals 19](#__RefHeading___Toc178244686)

[Table S8 Any or specific CLD before incident MALO while following patients with CeD, n (%) 24](#__RefHeading___Toc178244687)

[Table S9 Incident CLD and MALO in childhood-onset patients diagnosed with CeD before 2012 25](#__RefHeading___Toc178244688)

[Table S10 Sensitivity analyses of incident CLD and MALO in patients with CeD and their matched reference individuals 26](#__RefHeading___Toc178244689)

[Table S11 Characteristics of patients with CeD and their siblings, n (%) 27](#__RefHeading___Toc178244690)

[Table S12 Incident CLD and MALO in patients with CeD and their full siblings 28](#__RefHeading___Toc178244691)

[Table S13 Subgroup analyses of incident CLD and MALO in patients with CeD and their full siblings 29](#__RefHeading___Toc178244692)

[Table S14 Characteristics of CeD patients who had a follow-up biopsy within six months and five years after diagnosis, n (%) 34](#__RefHeading___Toc178244693)

[Table S15 Incident CLD and MALO in CeD patients who had a follow-up biopsy after diagnosis 35](#__RefHeading___Toc178244694)

[References 36](#__RefHeading___Toc178244695)

**Figure S1 Flow chart for study population selection**

**CeD: celiac disease; ESPRESSO: the Epidemiology Strengthened by histoPathology Reports in Sweden; HIV: human immunodeficiency virus.**


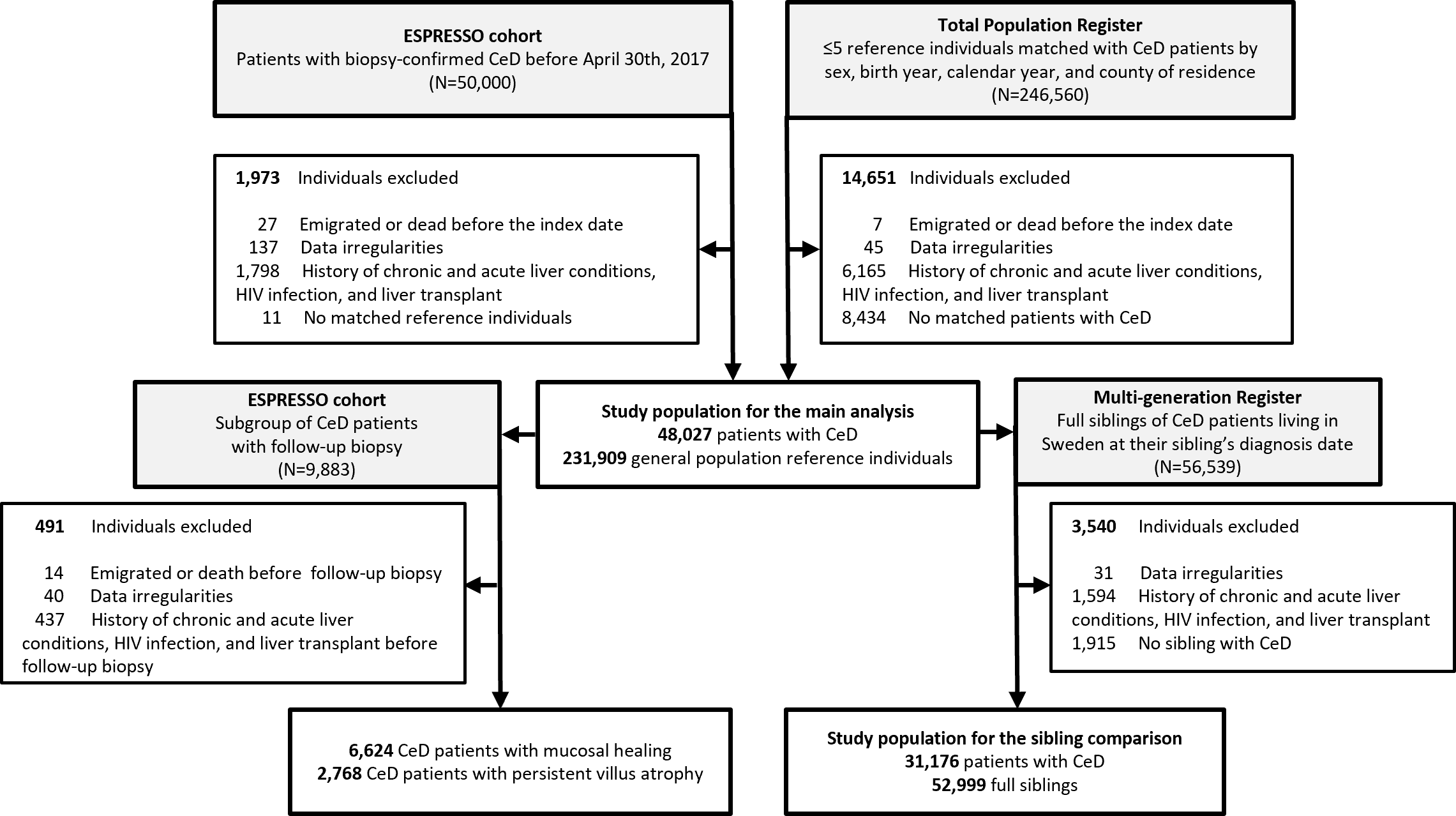


**Table S1a Synthesized or population-based evidence on the CLD risk in CeD**

| **Current study: CLD risk (primary outcome) and risk of specific CLD and MALO (secondary outcomes)** | | | | | | | | |
| --- | --- | --- | --- | --- | --- | --- | --- | --- |
| **First author, publication year,  place,  data source, study period, follow-up time** | **Sample size** | **Age at CeD diagnosis, years** | **Females, %** | **Number of outcomes in CeD** | **Incidence rate of outcome, per 100,000 person-years** | **First year of follow-up included? (“-” if not cohort study)** | **Covariates** | **Main findings** |
| Yao,  (current study), Sweden, Nationwide,  1969-2017 followed through 2021,  Median 16·0 years,  (IQR: 10·2-23·0) | Biopsy-confirmed CeD: 48,027 | Median 27·3 years,  all ages included | 62·7 | Any CLD: 649  Viral hepatitis: 134  MASLD: 131  Alcohol-related liver disease: 109 Autoimmune liver disease: 294  MALO: 580 | Any CLD: 79·4  Viral hepatitis: 16·3  MASLD: 16·4  Alcohol-related liver disease: 13·3 Autoimmune liver disease: 35·9  MALO: 65·2 | Included, excluded in sensitivity analysis | (Model 1)  1. birth year 2. sex 3. county of residence 4. calendar year  +  (Model 2)  5. country of birth 6. education level 7. number of healthcare visits 8. disease history of autoimmune diseases before the index date | Increased risk of any CLD in CeD.  aHR (same as below)=2·01 (95%CI: 1·82 to 2·22)  Driven by  Autoimmune liver disease: 4·86 (4·06 to 5·82)  MASLD: 2·54 (2·03 to 3·18)  Alcohol-related liver disease: 1·51 (1·20 to 1·90)  But not viral hepatitis:  0·89 (0·73 to 1·10)  In addition, higher risk of MALO: 1·54 (1·40 to 1·70) |
| **Meta-analyses** | | | | | | | | |
| **First author, publication year** | **Updated on** | | **No· of studies** | | **I^2^, %** | | **Pooled estimates (95% CI)** | |
| Aggarwal^1^, 2024 | 18 November 2022 | | Fatty liver: 10, of which 7 are longitudinal | | Fatty liver, before the GFD: 95; Fatty liver, after the GFD: 93 | | Fatty liver, before the GFD: prevalence in CeD (same as below)=18·2% (8·3% to 30·8%) Fatty liver, before the GFD, only longitudinal studies: 15·3% (5·6% to 28·5%) Fatty liver, after the GFD, only longitudinal studies: 29·1% (17·2% to 42·7%) | |
| Jena^2^, 2023 | 3 September 2020 (for CLD relevant to the current study) | | AIH: 12 PBC: 10 PSC: 8 NASH/NAFLD: 7 HBV: 6 HCV: 5 ASH: 4 | | AIH: 67 PBC: 22 PSC: 72 NASH/NAFLD: 0 HBV: 75 HCV: 36 ASH: 65 | | AIH: prevalence in CeD (same as below)=17·69% (10·14% to 29·05%) PBC: 7·82% (5·01% to 12·00%) PSC: 9·02% (3·62% to 20·70%) NASH/NAFLD: 6·43% (3·64% to 11·13%) HBV: 22·64% (7·10% to 52·86%) HCV: 21·07% (9·50% to 40·42%) ASH: 16·38% (5·16% to 41·33%) | |
| **Individual studies for specific CLD** | | | | | | | | |
| **Viral hepatitis** | | | | | | | | |
| **First author, publication year,  place,  data source, study period, follow-up time** | **Sample size** | **Age at CeD diagnosis, years** | **Females, %** | **Number of outcomes in CeD** | **Incidence rate of outcome, per 100,000 person-years** | **First year of follow-up included?** | **Covariates** | **Main findings** |
| Habash^3^, 2022, the US,  cases identified in  2009 and 2014 (NHANES database); 1998 and 2021 (single center, Mayo Clinic); 2010 and 2020  (REP, regional medical record data);  No follow-up for the cross-sectional study | 93 (NHANES)  3,568 (Mayo Clinic)  3,918 (REP) | aged ≤18 years:  26·88% of NHANES  14·46% in Mayo Clinic  16·05% in REP | 62/93 (=67%) in NHANES  2,690/3,918 (=69%) in the Mayo Clinic  2,389/3,568 (=67%) in REP | HBV  0 in NHANES  4 (0·11%) in Mayo Clinic  9 (0·23%) in REP | - | - | Not adjusted | In any of the three data sources, no increased risk of HBV infection was detected in CeD patients |
| **MASLD (previously nomenclated as NAFLD)^4^** | | | | | | | | |
| **First author, publication year,  place,  data source, study period, follow-up time** | **Sample size** | **Age at CeD diagnosis, years** | **Female, %** | **Number of outcomes in CeD** | **Incidence rate of outcome, per 100,000 person-years** | **First year of follow-up included?** | **Covariates** | **Main findings** |
| Hitawala^5^, 2023, the US,  multicenter in- and outpatient,  1999 to September 2022, No follow-up for the cross-sectional study | 136,735 | ≥65 years: 28·79%  Only adult patients (≥18 years) | 75·25 | NAFLD: 955 (0·7%) | - | - | NAFLD:  1. age (≥65 years) 2. gender 3. Caucasian race 4. diabetes mellitus (type 1 and type 2) 5. hypothyroidism 6. metabolic syndrome 7. obesity | NAFLD with type 1 diabetes: aOR=2·1 (95%CI: 1·96 to 2·25)  NAFLD with type 2 diabetes: aOR=2·92 (95%CI: 2·72 to 3·14) |
| Hitawala^6^ [in abstract], 2022, the US, multicenter in- and outpatient register study period not reported  No follow-up for the cross-sectional study | 137,010 | 28·87% aged ≥65 years | 75·21 | NAFLD  955 (0·7%) | - | - | Stepwise covariate inclusion, point estimates reported after adjusting for  1. Obesity 2. metabolic syndrome 3. type 2 diabetes mellitus | After multivariable adjustment, the odds of having NAFLD among patients with CeD, compared to non-CeD patients, was 3·21 (95%CI: 3·00 to 3·43) |
| Voss^7^, 2021, UK, nationwide biomarker database,  2006 to 2010 (diagnosis since 1996),  No follow-up for the cross-sectional study | 2,377 | Mean (SD) of enrolled age:  58·0 ± 7·8 (range 37 to 73) | 65 | NASH: 0·25% | - | - | 1. age 2. sex 3. body mass index 4. diabetes mellitus 5. mean alcohol consumption | NASH:  aOR=4·87 (95%CI: 2·16 to 11·00) |
| Reilly^8^, 2015, Sweden, nationwide in- and outpatient register, plus nationwide histopathology cohort, 1997 to 2009, median follow-up: 10 years | Biopsy-confirmed CeD 26,816 | Median age 28 years  42·8% aged ≤19; 16·8% aged >60 years | 62·5 | NAFLD  53 (0·2%) | 21 in CeD; 6 in general population reference individuals | Included, excluded in sensitivity analysis | Multivariate analysis:  1. country of birth (Nordic vs. not Nordic),  2. self or parental educational level,  3. socioeconomic status at the time of biopsy | Increased risk of NAFLD in patients with CeD aHR=2·8 (95%CI: 2·0 to 3·8).  The point estimate for the relative risk of NAFLD was higher in males than in females as well as in childhood-onset CeD than in CeD diagnosed ≥40 years, while both did not reach statistical significance.  After excluding the first year of follow-up, the relative risk of being diagnosed with NAFLD in patients with CeD was aHR=2·5 (95%CI: 1·8 to 3·7) |
| **Alcohol-related liver disease** | | | | | | | | |
| **First author, publication year,  place,  data source, study period, follow-up time** | **Sample size** | **Age at CeD diagnosis, years** | **Female, %** | **Number of outcomes in CeD** | **Incidence rate of outcome, per 100,000 person-years** | **First year of follow-up included?** | **Covariates** | **Main findings** |
| Ludvigsson^9^ *, 2007, Sweden,  nationwide inpatient register,  1964 to 2003, follow-up time not reported | 13,818 | Median (range):  2 (0-94);  0-15 years: 67·4%; ≥16: 32·6% | 58·7 | Not reported | Not reported | Excluded; included in sensitivity analysis | 1. year of birth (age) 2. sex 3. calendar period  only in sensitivity analysis  4. presence of diabetes mellitus 5. socioeconomic status 6. use of alcohol | (including first follow-up year)  alcohol-induced liver disease: aHR=2·13 (95%CI: 1·28 to 3·53) |
| **Autoimmune liver disease (including AIH, PBC, PSC)** | | | | | | | | |
| **First author, publication year,  place,  data source, study period, follow-up time** | **Sample size** | **Age at CeD diagnosis, years** | **Female, %** | **Number of outcomes in CeD** | **Incidence rate of outcome, per 100,000 person-years** | **First year of follow-up included?** | **Covariates** | **Main findings** |
| Conrad^10^, 2023, UK,  nationwide primary and secondary care database,  1 Jan 2000 to 30 June 2019,  (1 Jan 1985 for disease history retrospection),  mean follow-up for all individuals with incident autoimmune diseases: 6·17 years | 31,447 | Median (IQR): 45 (26-62) | Not reported (IRR for all incident CeD: female vs male: 1·95 (95% CI 1·88 to 2·01) | Not reported | Not reported | Excluded | (negative binomial regression models)  1. calendar year 2. age 3. sex 4. socioeconomic status 5. region | Significantly increased risk of incident PBC/PSC in patients with CeD  PBC/PSC ** in patients with CeD: aIRR=5·0 (95%CI: 3·9 to 6·4) |
| Hitawala^5^, 2023, the US,  multicenter in- and outpatient,  1999 to September 2022, No follow-up for the cross-sectional study | 136,735 | ≥65 years: 28·79%  Only adult patients (≥18 years) | 75·25 | AIH: 440 (0·32%)  PBC: 200 (0·15%) PSC: 5 (0·004%) NAFLD: 955 (0·7%) | - | - | AIH, PBC, PSC:  1. age (≥65 years) 2. gender 3. Caucasian race 4. presence of anti-tissue transglutaminase antibody (anti-TTG) | AIH: aOR (same as below)=7·06 (95%CI: 6·32 to 7·89) adjusting for anti-TTG: 4·79 (95%CI: 3·88 to 5·92)  PBC: 4·16 (95%CI: 3·46 to 5·00) adjusting for anti-TTG: 9·22 (95%CI: 7·03 to 12·1)  Multivariable analysis on OR of PSC was not performed due to a low sample size |
| Voss^7^, 2021, UK, nationwide biomarker database,  2006 to 2010 (diagnosis since 1996),  No follow-up for the cross-sectional study | 2,377 | Mean (SD) of enrolled age:  58·0 ± 7·8 (range 37 to 73) | 65 | AIH: 0·25% cholangitis: 0·29% | - | - | 1. age 2. sex 3. body mass index 4. diabetes mellitus 5. mean alcohol consumption | AIH: aOR=5·50 (95%CI: 2·43 to 12·43)  Cholangitis: - not significantly increased odds in patients with CeD compared to the reference individuals who had none of CeD or inflammatory bowel disease. |
| Ludvigsson^9^ *, 2007, Sweden,  nationwide inpatient register,  1964 to 2003, follow-up time not reported | 13,818 | Median (range):  2 (0-94);  0-15 years: 67·4%; ≥16: 32·6% | 58·7 | (after excluding one follow-up year)  PSC: 26 (0·188%)  PBC: 7 (0·051%) | Not reported | Excluded; included in sensitivity analysis | 1. year of birth (age) 2. sex 3. calendar period  + (sensitivity analysis)  4. presence of diabetes mellitus 5. socioeconomic status 6. use of alcohol | (after excluding one follow-up year)  PSC: aHR (same as below)=4·46 (95%CI: 2·50 to 7·98) PBC: 10·16 (95%CI: 2·61 to 39·49)  (including first follow-up year)  PSC: 13·62 (95%CI: 4·38 to 42·33) PBC: 5·93 (95%CI: 3·68 to 9·56)  Point estimates increased (all became significant) after including the first follow-up year; while the point estimates decreased when using inpatient reference individuals.  Additionally adjusting for diabetes mellitus, socioeconomic status, and use of alcohol did not affect the risk estimates notably. |
| Lawson^11^, 2005,  UK,  nationwide primary care database,  cases identified from June 1987 to April 2002, No follow-up for cross-sectional study | 4,732 | 24·2% aged ≤25 years  (reported in the same cohort)^12^ | 65·4  (reported in the same cohort)^12^ | PBC: 8 (0·17%)  PSC: 2 (0·04%) | - | - | Matching variables: 1. age 2. sex 3. general practice 4. follow-up time  aOR also adjusted for smoking status | Both PBC and PSC had a higher prevalence in patients with CeD than to the age, sex, general practice, and follow-up time-matched controls.  There is an increased occurrence of PBC in patients with CeD: aOR=3·69 (95%CI: 1·48 to 9·23) |
| Sørensen^13^, 1999, Denmark, Danish and Swedish nationwide inpatient register,  Danish cohort: 1 January 1977 to 31 December 1992; followed for a median (IQR) of 9·1 (4·5 to 13·3) years;  Swedish cohort: 1987 to 1996; followed for 5·1 (2·5 to 7·7) years | 896 in Danish cohort and 7,735 in Swedish cohort | Not reported | Not reported | Not reported | Not reported | Included | Population standardized (details not reported) | Significantly increased risk of PBC was observed both in the Danish cohort and the Swedish cohort of patients with CeD.  In Danish cohort: Standardized IRR=27·6 (95%CI: 2·9 to 133·5)  In Swedish cohort: Standardized IRR=25·1 (95%CI: 15·7 to 37·9) |
| Kingham^14^, 1998, UK, regional primary and secondary care register, cases observed from 1984 to 1996; No follow-up for cross-sectional study | 143 | Not reported | 73·4 | PBC: 4 (2·80%) | - | - | - | Prevalence of PBC in patients with CeD was 2·80% |
| **Individual studies for components of MALO (a composite endpoint for compensated or decompensated cirrhosis, HCC, liver transplantation, and liver-related death)** | | | | | | | | |
| **First author, publication year,  place,  data source, study period, follow-up time** | **Sample size** | **Age at CeD diagnosis, years** | **Female, %** | **Number of outcomes in CeD** | **Incidence rate of outcome, per 100,000 person-years** | **First year of follow-up included?** | **Covariates** | **Main findings** |
| Lebwohl^15^, 2022, nationwide in- and outpatient register, plus nationwide histopathology cohort, 1965 to 2016, median follow-up: 11·3 years | Biopsy-confirmed CeD 47,241 | Median (IQR):  26·0 (7·7-50·9) | 62·2 | All biliary/liver cancer (primary or secondary cancer of HCC or biliary malignancy):  115 (0·2%) | 20 vs 10 | Included, excluded in the sensitivity analysis | Model 1  1. age 2. sex 3. county 4. calendar period  +  Model 2  5. education  6. country of birth (Nordic vs. not Nordic)  7. time-dependent comorbidities (type 1 diabetes, autoimmune thyroid disease, rheumatoid arthritis, and inflammatory bowel disease) | All biliary/liver cancer:  Model 1: aHR=1·80 (95%CI: 1·44 to 2·25)  Model 1, excluding first year of follow-up: aHR=1·61 (95%CI: 1·26 to 2·05)  Model 2: aHR=1·45 (95%CI: 1·13 to 1·86) |
| Voss^7^, 2021, UK, nationwide biomarker database,  2006 to 2010 (diagnosis since 1996),  No follow-up for the cross-sectional study | 2,377 | Mean (SD) of enrolled age:  58·0 ± 7·8 (range 37 to 73) | 65 | Cirrhosis: 0·55%;  HCC: 0·17% | - | - | 1. age 2. sex 3. body mass index 4. diabetes mellitus 5. mean alcohol consumption | Cirrhosis: aOR=3·59 (95%CI: 2·11 to 6·10)  Chronic cirrhosis: aOR=8·51 (95%CI: 3·97 to 18·22)  HCC: aOR=4·79 (95%CI: 1·77 to 12·96) |
| Ludvigsson^9^ *, 2007, Sweden,  nationwide inpatient register,  1964 to 2003, follow-up time not reported | 13,818 | Median (range):  2 (0-94);  0-15 years: 67·4%; ≥16: 32·6% | 58·7 | (after excluding one follow-up year)  Ascites: 13 (0·094%)  Liver failure, extended: 45 (0·326%)  Liver failure restricted: 7 (0·051%)  Liver cirrhosis/liver fibrosis: 23 (0·166%)  Liver transplantation: 1 (0·007%) | Not reported | Excluded; included in sensitivity analysis | 1. year of birth (age) 2. sex 3. calendar period  only in sensitivity analysis  4. presence of diabetes mellitus 5. socioeconomic status 6. use of alcohol | (after excluding one follow-up year)  Ascites: aHR (same as below)=1·31 (95%CI: 0·70 to 2·46) Liver failure, extended: 3·30 (95%CI: 2·22 to 4·88) Liver failure restricted: 3·62 (95%CI: 1·35 to 9·75) Liver cirrhosis/liver fibrosis: 2·23 (95%CI: 1·34 to 3·72) Liver transplantation: 1·07 (95%CI: 0·12 to 9·62)  (including one follow-up year) Ascites: 2·87 (95%CI: 1·79 to 4·60) Liver failure, extended: 7·58 (95%CI: 5·56 to 10·34) Liver failure restricted: 10·08 (95%CI: 4·56 to 22·29) Liver cirrhosis/liver fibrosis: 6·27 (95%CI: 4·32 to 9·09) Liver transplantation: 5·47 (95%CI: 1·46 to 20·48)  Point estimates increased (all became significant) after including the first follow-up year; while the point estimates decreased when using inpatient reference individuals.  Additionally adjusting for diabetes mellitus, socioeconomic status, and use of alcohol did not affect the risk estimates notably. |

* Also included in the meta-analysis by Jena et al.^2^

** Although being reported as PBC, cases with PSC may have been included in the study by Conrad et al. (**Table S3**).^10^

AIH: autoimmune hepatitis; ASH: alcoholic steatohepatitis; GFD: gluten-free diet; PBC: primary biliary cholangitis; PSC: primary sclerosing cholangitis; CeD: celiac disease; CI: confidence interval; CLD: chronic liver disease; HBV/HCV: hepatitis B/C virus infection; HCC: hepatocellular carcinoma; (a)HR: (adjusted) hazard ratio; (a)IRR: (adjusted) incidence rate ratio; IQR: interquartile range; MALO: major adverse liver outcomes; MASLD: metabolic dysfunction–associated steatotic liver disease; NAFLD: nonalcoholic fatty liver disease; NASH: nonalcoholic steatohepatitis; NHANES: National Health and Nutrition Examination Survey; REP: Rochester Epidemiology Project; SD: standard deviation; (a)OR: (adjusted) odds ratio

Table S1b Synthesized or population-based evidence on the CeD risk in CLD

| **Meta-analyses** | | | | | | | | |
| --- | --- | --- | --- | --- | --- | --- | --- | --- |
| **First author, publication year** | **Updated to** | | **No. of studies** | | **I^2^, %** | | **Pooled estimates (95% CI)** | |
| Yoosuf^16^, 2023 | January 2022 | | Cryptogenic cirrhosis:  9 for both seroprevalence and biopsy-confirmed CeD All-cause cirrhosis:  7 for CeD seroprevalence 5 for biopsy-confirmed CeD | | Cryptogenic cirrhosis:  91 for CeD seroprevalence  30 for biopsy-confirmed CeD All-cause cirrhosis:  90 for CeD seroprevalence 97 for biopsy-confirmed CeD | | Biopsy-confirmed CeD in  cryptogenic cirrhosis: prevalence (same as below)=4·55% (95%CI: 2·19% to 7·51%) all-cause cirrhosis: 0·76% (95%CI: 0 to 3·43%)  CeD seroprevalence in cryptogenic cirrhosis: 15·31% (95%CI: 4·91% to 29·50%) all-cause cirrhosis: 14·19% (95%CI: 3·86 to 28·81%) | |
| Haggård^17^, 2021 | 7 February 2020 | | AIH: 8 for biopsy-confirmed CeD and 15 for biopsy- or seropositive CeD | | 0·0 for biopsy-confirmed CeD;  66·8 for biopsy-confirmed or seropositive CeD· | | Biopsy-confirmed CeD in AIH: prevalence=3·5% (95%CI: 1·6% to 5·3%)  Biopsy-confirmed or seropositive CeD in AIH: prevalence=2·9% (95%CI: 2·1% to 3·8%) | |
| Vajro^18^, 2013 | From 1977 to May 2012 | | AIH: 3 for biopsy-confirmed pediatric (0-18 years) CeD | | 68·6 for biopsy-confirmed pediatric CeD | | Biopsy-confirmed pediatric CeD in AIH: prevalence=8·51 (95%CI: 3·28 to 22·05) | |
| **Individual studies for specific CLD** | | | | | | | | |
| **First author, publication year,  place,  data source, study period, follow-up time** | **Sample size** | **Age at CLD diagnosis, years** | **Female, %** | **Number of CeD in CLD** | **Incidence rate of CeD, per 100,000 person-years** | **First year of follow-up included?** | **Covariates** | **Main findings** |
| Båve^19^, 2024, Sweden, nationwide cohort, from 2001 through 2019, median (IQR) follow-up of 12·6 (7·5 to 19) years: | 1,378 with PSC | Mean (SD): 33.00 (14·39) | 31.86 | 18 (1.30%) | Not reported | Included | Matching variables: 1. gender 2. age 3. residency | There was a higher odds of incident CeD in patients with PSC compared to PSC-free individuals: OR=4·36 (95%CI: 2·44 to 7·49); |
| Roderburg^20^, 2022, Germany, nationwide primary care (general practice) register,  January 2000 to December 2015, follow-up up to ten years | 57,336 with NAFLD * | Mean (SD): 60·3 (13·9)  aged ≥18 years | 47·8 | 0·104% | 11·2 (compared to 7·5 for non-NAFLD) | Included | Matching variables: 1. gender 2. age 3. index year  No covariate adjustment involved | There was a higher risk of incident CeD in patients with NAFLD compared to non-NAFLD patients: aHR=1·50 (95%CI: 0·95 to 2·36);  The relative risk of CeD was significantly higher in patients aged 18 to 50 years aHR=2·79 (95%CI: 1·12 to 6·94) and among male patients aHR=2·02 (95%CI: 1·01 to 4·04) |
| Renno A^21^, 2021, the US, nationwide administrative database for hospitalized patients, case records from 2016, no follow-up for the cross-sectional study | 10,740 with NASH (baseline for 10,950 cases) | Mean (SD): 61·21 (13·92) | 61·28 | 75 (0·68%) | - | - | - (Unadjusted, since CeD was one of the adjusted comoridities) | OR=3·86 (95%CI: 1·91 to 7·81) |
| **Individual studies for components of MALO (a composite endpoint for compensated or decompensated cirrhosis, HCC, liver transplantation, and liver-related death)** | | | | | | | | |
| **First author, publication year,  place,  data source, study period, follow-up time** | **Sample size** | **Age at MALO diagnosis, years** | **Female, %** | **Number of outcomes in MALO** | **Incidence rate of outcome, per 100,000 person-years** | **First year of follow-up included?** | **Covariates** | **Main findings** |
| Grønbæk^22^, 2015, Denmark, nationwide in- and outpatient register, 1 January 1977 to 1 March 2010, length of follow-up not reported | 24,679 with *alcoholic* cirrhosis | Not reported | Not reported | 18 | Not reported | Included | Matching variables: 1. sex 2. birth week Adjusted covariates: 3. hospitalization frequency within one year before inclusion | incident CeD in patients with *alcoholic* cirrhosis, compared to age- and sex-matched controls: aIRR=5·12 (95%CI: 2·58 to 10·16) |

AIH: autoimmune hepatitis; CeD: celiac disease; CI: confidence interval; CLD: chronic liver disease; (a)IRR: (adjusted) incidence rate ratio; IQR: interquartile range; MALO: major adverse liver outcomes; NAFLD: nonalcoholic fatty liver disease; NASH: nonalcoholic steatohepatitis; PSC: primary sclerosing cholangitis; SD: standard deviation; OR: odds ratio.

* Later nomenclated as MASLD^4^

| **Table S2 SNOMED codes defining CeD ^a^** | | |
| --- | --- | --- |
| **Disease/Condition** | **Topographic codes** | **SNOMED codes ^b^** |
| Celiac disease (villus atrophy, VA) | Duodenum: T64 (all);  Jejunum and early duodenum: T65, T65000 and T651 (excluding T652-9) | Celiac diagnosis: D6218, D62180, D62188, D6218X, D6218Y;  Marsh 3: M58, M5800, M58000, M58001, M58005, M58006, M58007 |
| Mucosal healing | T64, T65, T65000, T651 | Marsh 0: M00100, M00110,  Marsh 1-2: M40000, M41000, M42000, M43000, M47000, M47170 |
| CeD: celiac disease; SNOMED: Systematized Nomenclature of Medicine;  ^a^ Diagnosis criteria: ≥1 SNOMED code for VA in the small intestine other than the ileum (PPV: 95% for biopsy samples between 1969 and 2008, and 99% for biopsy samples between 2009 and 2017).^23,24^  ^b^ In SNOMED codes, diagnostic codes that were listed under morphology in pathology registers starts with “D”. For example D6218 was the diagnostic code for CeD. Meanwhile, codes that start with “M” (suggestive of inflammation but was not specific) in the table refer to all SNOMED codes starting with the respective code, which shall be accompanied by a topographic code.  The presence of persistent VA (Marsh 3) or mucosal healing (Marsh 0-2) was determined based on the follow-up biopsy result within six months and five years after the first biopsy that indicates Marsh 3 small intestinal VA. | | |

Table S3 Definitions of exclusion criteria

| **Exclusion** | **Data source** | **ICD-8 (1969-1986)** | **ICD-9 (1987-1996)** | **ICD-10 (1997-)** | **ICD-7 (for the Cancer register)** |
| --- | --- | --- | --- | --- | --- |
| Any primary liver cancer | NPR, the Cancer Register | 155,0; 156 | 155 | C22 | 155,0; 156 |
| Infectious hepatitis | NPR | 070; 999,20 | 070 | B15-19; B008; B251; K770 | - |
| MASLD (including MASH) ^a^ | NPR | 571,01; 571,9 | 571W | K758; K760 | - |
| Alcohol abuse/misuse or alcohol-related liver disease, including alcohol-related cirrhosis | NPR | 261,00; 262,00; 280,00; 281,00; 291; 291,1; 303; 307,00; 307,10; 307,99; 322; 571,00; 571,01; 581,10; 583,10; 979; 980,00; 980,01; 980,98; 980,99 | 291; 294A; 303; 305A; 357F; 425F; 535D; 571A-D; 760W; 790D; 977D; 980A; 980X; V97B | E244; F10; G312; G621; G721; I426; K292; K70; K860; O354; R780; T510; T518; T519; X65; Y15; Y573; Y90; Y91; Z502; Z714; Z712 | - |
| Other abuse- and drug-related diagnoses | NPR | 571,0; | 571A-571D | F11-F19 | - |
| Drug-induced liver disease | NPR | - | 573D | K71 | - |
| Congestive hepatopathy | NPR | - | 573A | K761 | - |
| Cirrhosis, compensated or decompensated | NPR | 456,00; 570-573; 785,3 | 456A-456C; 570-572; 789F | I850; I859; I864; I982; I983; R18; K703; K704; K72; K74; K76 | - |
| Autoimmune liver diseases (including AIH, PBC, and PSC) | NPR | 571,9x; 573,0x; 575,05 | 571E; 573D; 571G; 576B | K743-K745; K754; K830A | - |
| Hepatic veno-occlusive disease (Budd-Chiari) | NPR | - | 453A | I82 | - |
| Liver abscess | NPR | 572 | 572A | K750; A064 | - |
| HIV infection | NPR | - | 279K; 079J | B20-B24; F024; Z219; Z717; F02.4; O987; R75 | - |
| Hemochromatosis | NPR | 273,2 | 275A | E831 | - |
| Wilson’s disease | NPR | 273,3 | 275B | E830 | - |
| Alpha-1 antitrypsin deficiency | NPR | - | 277G | E880 | - |
| Glycogen storage disease | NPR | - | 271W; 271X | E74 | - |
| Liver transplantation ^b^ | NPR | KVÅ for procedure (1987-1996): 5200-5299 | V42H; KVÅ for procedure (1987-1996): 5200-5299 | ICD-10: Z944; KVÅ for procedure (1997-): JJC; DJ005; DJ006 | - |

AIH: autoimmune hepatitis; CeD: celiac disease; HIV: human immunodeficiency virus; ICD: International Classification of Disease; MASH: Metabolic dysfunction-associated steatohepatitis; MASLD: metabolic dysfunction-associated steatotic liver disease; NPR: National Patient Register; PBC: primary biliary cholangitis; PSC: primary sclerosing cholangitis

^a^ MASLD was formerly nomenclated as non-alcoholic fatty liver disease.^4^ A Swedish study described that over 99·5% of patients with non-alcoholic fatty liver disease also met the criteria for MASLD.^25^

^b^ Liver transplantation was identified through listed procedural codes.

Table S4 Definitions of outcomes, comorbidities, and medications

| **Outcomes** | **Data source** | **ICD-8**  **(1969-1986)** | **ICD-9**  **(1987-1996)** | **ICD-10**  **(1997-)** | **ICD-7 (for the Cancer register)** | **ATC** |
| --- | --- | --- | --- | --- | --- | --- |
| Primary outcome | | | | | | |
| Any CLD | NPR | Identified through the codes of different etiologies below | | | | |
| Secondary outcomes: specific CLD and MALO | | | | | | |
| CLD by etiology | | | | | | |
| Viral hepatitis (i.e., hepatitis virus A, B, C, D, and E infection) | NPR | 070; 999,20 | 070 | B15-19 | - | - |
| MASLD (including MASH) ^a^ | NPR | 571,01; 571,9 | 571W | K758; K760 | - | - |
| Alcohol-related liver disease | NPR | 571,00; 571,01 | 571A-D | K700; K701; K702; K703; K704; K709 | - | - |
| Autoimmune liver diseases (including AIH, PBC, and PSC) | NPR | 571,9; 573,0; 575,05 | 571E; 573D; 571G; 576B | K743; K745; K754; K830A | - | - |
| AIH | NPR | 571,9; 573,0; | 571E; 573D | K754 |  |  |
| PBC | NPR | 575,05 without prior 563 | 571G | K743; K745; |  |  |
| PSC | NPR | 575,05 + 563 | 576B | K830A |  |  |
| MALO | | | | | | |
| Cirrhosis, compensated or decompensated | NPR | 571,00; 571,90; 571,98; 571,99 (cirrhosis);  456,00 (esophageal/gastric varices);  785,3 (ascites);  570 (acute or subacute liver failure);  573 (chronic or unspecified liver failure) | 571C; 571F (cirrhosis)  572D (portal hypertension)  572C (hepatic encephalopathy)  456A-456C (esophageal/gastric varices, bleeding or non-bleeding)  789F (ascites)  572E (hepatorenal syndrome)  570 (acute or subacute liver failure);  572W (chronic or unspecified liver failure) | K703; K717; K746 (cirrhosis)  K766 (portal hypertension)  K767 (hepatorenal syndrome)  R18 (ascites)  I850; I859; I864; I982; I983 (bleeding esophageal/gastric varices, bleeding or non-bleeding)  K704 (liver failure caused by alcohol);  K720 (acute or subacute liver failure);  K721; K729 (chronic or unspecified liver failure) | - | - |
| HCC | NPR, the Cause of Death Register, the Cancer Register | 155,0 | 155A | C220 | 155,0 | - |
| Liver transplantation | NPR | KVÅ for procedure (1987-1996): 5200-5299 | V42H; KVÅ for procedure (1987-1996): 5200-5299 | Z944; KVÅ for procedure (1997-): JJC; DJ005; DJ006 | - | - |
| Liver-related death | the Cause of Death Register, TPR | 070; 999,20; 456,00; 570-576; 155,0; 785,3; 156; (procedural codes) 5200-5299 | 070; 571-573; 155; 456A-456C; 789F; V42H; (procedural codes) 5200-5299 | B15-B19; K70-K77; I850; I859; I864; I982; I983; R18; C22; Z944; (procedural codes) JJC; DJ005; DJ006 | - | - |
| **Comorbidities** | **Data source** | **ICD-8**  **(1969-1986)** | **ICD-9**  **(1987-1996)** | **ICD-10**  **(1997-)** | **ICD-7**  **(for the Cancer register)** | **ATC** |
| Autoimmune diseases | | | | | | |
| Systemic lupus erythematosus/cutaneous lupus erythematosus | NPR | 734,1 | 710A | M32; L931 | - | - |
| Vitiligo | NPR | 709,05 | - | L80 | - | - |
| Psoriasis | NPR | 696,0; 696,1 | 696A; 696B | L40 | - | - |
| Alopecia areata | NPR | 704,00 | - | L63 | - | - |
| Systemic sclerosis | NPR | 734,00-734,19 | 710B | M34 | - | - |
| Dermatomyositis or juvenile dermatomyositis | NPR | 716,00 | 710D | M33 except M332 | - | - |
| Polymyositis | NPR | 716,10 | 710E | M332 | - | - |
| Type 1 diabetes | NPR | 250 (only if the first diagnosis ≤30 years ^b^) | 250 (only if the first diagnosis ≤30 years ^b^) | E10 | - | - |
| Autoimmune thyroid disease | NPR | 242,00; 242,09; 244; 245,02; 245,03 | 242A; 242X; 244X; 245C; 245W | E035; E039; E050; E055; E059; E063; E065 | - | - |
| Addison’s disease | NPR | 255,10 | 255E | E271; E272 | - | - |
| Myasthenia gravis | NPR | 733,00 | 358A | G700 | - | - |
| Inflammatory bowel disease | NPR | 563; 563,0; 563,00; 563,1; 563,10; 563,9; 563,98; 563,99; 569,02; 569,04 | 555; 556 | K50; K51; K523 | - | - |
| Sjögren syndrome | NPR | 734,90 | 710C | M350 | - | - |
| Lambert-Eaton syndrome | NPR | - | - | G731 | - | - |
| Rheumatoid arthritis | NPR | 712,10; 712,20; 712,38; 712,39 | 714A; 714B; 714C; 714W; 719D | M05; M060; M062; M063; M068; M069; M123 | - | - |
| Juvenile arthritis | NPR | 712,0 | 714D | M08 | - | - |
| Multiple sclerosis | NPR | 340,99 | 340 | G35 | - | - |
| Ankylosing spondylitis | NPR | 712,40 | 720A | M45 | - | - |
| Spondyloarthritis | NPR | 713,13; 726,99 | 720A; 696A; 713B; 099D; 711A; 720B; 720C; 720W | M45; M081; L405; M070-M076; M0912; M020-M023; M028-M029; M460; M461; M468; M469 | - | - |
| Sarcoidosis | NPR | 135 | 135 | D86; G532; M633 | - | - |
| Metabolic-related diseases, only for subgroup analysis | | | | | | |
| Hypertension | NPR, PDR | 400-404 | 401-405 | I10; I119; I12-I15; | - | C02, C03AA-AB, C03BA, C03CA, C03DA, C03EA, C08CA, C08DA, C08DB, C09A, C09BA, C09BB, C09CA, C09DA, C09DB01 |
| Diabetes mellitus | NPR, PDR | 250 | 250 | E10-E14; O24 | - | A10 |
| Obesity | NPR | 277 | 278A; 278B | E65-E66 | - | - |
| Dyslipidemia | NPR, PDR | 279 | 272 | E78 | - | ATC codes for lipid-lowering medications: C10AA, C10AB, C10AC, C10AD, C10AX01-14 |

AIH: autoimmune hepatitis; ATC: anatomical therapeutic chemical; CeD: celiac disease; CLD: chronic liver disease; HCC: hepatocellular carcinoma; ICD: International Classification of Disease; MALO: major adverse liver outcomes; MASH: metabolic dysfunction-associated steatohepatitis; MASLD: metabolic dysfunction-associated steatotic liver disease; NPR: National Patient Register; PBC: primary biliary cholangitis; PDR: Prescribed Drug Register; PSC: primary sclerosing cholangitis; TPR: Total Population Register;

^a^ MASLD was formerly nomenclated as non-alcoholic fatty liver disease.^4^ A Swedish study described that over 99·5% of patients with non-alcoholic fatty liver disease also met the criteria for MASLD.^25^

^b^ The algorithm for identifying the diagnosis of type 1 diabetes via ICD-8 and ICD-9 was proved to have a high positive predictive value (>95%).^26^

| **Table S5 Incident CLD and MALO in patients with CeD and their matched reference individuals** | | | | | |
| --- | --- | --- | --- | --- | --- |
|  | **No. of events (IR, per 100,000 person-years)** | | **IR difference (95%CI), per 100,000 person-years** | **HR (95%CI)** | |
|  | **Patients** | **References** |  | **Model 1 ^a^** | **Model 2 ^b^** |
| Primary outcome |  |  |  |  |  |
| Any CLD | 649 (79·4) | 1571 (39·5) | 40·0 (33·6 to 46·4) | 2·16 (1·97 to 2·38) | 2·01 (1·82 to 2·22) |
| Secondary outcomes: specific CLD and MALO | | | | | |
| Viral hepatitis | 134 (16·3) | 731 (18·3) | -2·0 (-5·1 to 1·1) | 0·89 (0·73 to 1·08) | 0·89 (0·73 to 1·10) |
| MASLD | 131 (16·4) | 254 (6·5) | 9·9 (7·0 to 12·8) | 2·79 (2·24 to 3·47) | 2·54 (2·03 to 3·18) |
| Alcohol-related liver disease | 109 (13·3) | 363 (9·1) | 4·2 (1·5 to 6·8) | 1·60 (1·27 to 2·00) | 1·51 (1·20 to 1·90) |
| Autoimmune liver disease | 294 (35·9) | 271 (6·8) | 29·1 (24·9 to 33·3) | 5·84 (4·91 to 6·95) | 4·86 (4·06 to 5·82) |
| AIH | 123 (15·0) | 113 (2·8) | 12·2 (9·5 to 14·9) | 5·72 (4·37 to 7·50) | 5·14 (3·89 to 6·79) |
| PBC | 56 (6·8) | 92 (2·3) | 4·5 (2·7 to 6·4) | 3·24 (2·29 to 4·58) | 3·12 (2·18 to 4·45) |
| PSC | 139 (16·9) | 85 (2·1) | 14·8 (12·0 to 17·7) | 9·03 (6·78 to 12·02) | 6·32 (4·67 to 8·56) |
| MALO | 580 (70·7) | 1795 (45·0) | 25·7 (19·6 to 31·8) | 1·68 (1·53 to 1·85) | 1·54 (1·40 to 1·70) |
| Cirrhosis, compensated or decompensated | 507 (61·8) | 1537 (38·5) | 23·3 (17·5 to 29·0) | 1·73 (1·56 to 1·92) | 1·60 (1·44 to 1·78) |
| HCC | 59 (7·2) | 190 (4·8) | 2·4 (0·5 to 4·4) | 1·55 (1·15 to 2·10) | 1·40 (1·03 to 1·91) |
| Liver transplantation | 25 (3·0) | 35 (0·9) | 2·2 (0·9 to 3·4) | 3·36 (1·92 to 5·88) | 2·66 (1·48 to 4·79) |
| Liver-related death | 120 (14·6) | 417 (10·4) | 4·2 (1·4 to 6·9) | 1·44 (1·17 to 1·79) | 1·38 (1·11 to 1·72) |
| AIH: autoimmune hepatitis; CeD: celiac disease; CI: confidence interval; CLD: chronic liver disease; HCC: hepatocellular carcinoma; HR: hazard ratio; IR: incidence rate; MALO: major adverse liver outcomes; MASLD: metabolic dysfunction-associated steatotic liver disease; PBC: primary biliary cholangitis; PSC: primary sclerosing cholangitis | | | | | |
| ^a^ Model 1: conditioned on the matching variables (birth year, sex, county of residence, and calendar year of index date).  ^b^ Model 2: model 1+further adjusted for country of birth, educational attainment, number of healthcare visits between two years and six months before the index date, and the history of autoimmune diseases. | | | | | |

**Table S6** Cumulative incidence difference (95%CI) of incident CLD and MALO during follow-up in individuals with CeD, compared with their matched reference individuals

| **Outcomes** | **Difference in standardized cumulative incidence in percentages (95%CI), years since the index date** ^a^ | | | |
| --- | --- | --- | --- | --- |
|  | **1 year** | **5 years** | **10 years** | **25 years** |
| Primary outcome | | | | |
| Any CLD | 0·09 (0·06 to 0·11) | 0·26 (0·21 to 0·32) | 0·41 (0·33 to 0·49) | 0·91 (0·72 to 1·10) |
| Secondary outcomes: specific CLD and MALO | | | | |
| Viral hepatitis | 0·008 (-0·006 to 0·02) | 0·002 (-0·03 to 0·03) | -0·02 (-0·06 to 0·01) | -0·05 (-0·14 to 0·03) |
| MASLD | 0·02 (0·007 to 0·03) | 0·07 (0·04 to 0·09) | 0·11 (0·08 to 0·15) | 0·29 (0·15 to 0·43) |
| Alcohol-related liver disease | 0·01 (0·001 to 0·02) | 0·04 (0·01 to 0·06) | 0·05 (0·02 to 0·08) | 0·09 (-0·007 to 0·19) |
| Autoimmune liver disease | 0·05 (0·03 to 0·07) | 0·16 (0·13 to 0·20) | 0·28 (0·23 to 0·33) | 0·60 (0·49 to 0·71) |
| AIH | 0·02 (0·009 to 0·03) | 0·07 (0·04 to 0·09) | 0·11 (0·08 to 0·15) | 0·28 (0·20 to 0·35) |
| PBC | 0·02 (0·006 to 0·03) | 0·04 (0·02 to 0·06) | 0·05 (0·02 to 0·07) | 0·13 (0·06 to 0·02) |
| PSC | 0·02 (0·006 to 0·02) | 0·06 (0·04 to 0·08) | 0·13 (0·10 to 0·16) | 0·25 (0·19 to 0·32) |
| MALO | 0·08 (0·06 to 0·11) | 0·19 (0·14 to 0·24) | 0·25 (0·18 to 0·33) | 0·68 (0·41 to 0·95) |
| Cirrhosis, compensated or decompensated | 0·07 (0·05 to 0·10) | 0·17 (0·12 to 0·22) | 0·24 (0·17 to 0·30) | 0·66 (0·39 to 0·93) |
| HCC | 0·007 (-0·002 to 0·002) | 0·02 (-0·001 to 0·03) | 0·02 (-0·004 to 0·05) | 0·06 (-0·02 to 0·14) |
| Liver transplantation | -0·0002 (-0·001 to 0·0005) | 0·005 (-0·002 to 0·01) | 0·02 (0·003 to 0·03) | 0·04 (0·01 to 0·07) |
| Liver-related death | 0·006 (-0·002 to 0·01) | 0·02 (0·002 to 0·05) | 0·04 (0·002 to 0·07) | 0·16 (0·02 to 0·29) |
| AIH: autoimmune hepatitis; CeD: celiac disease; CI: confidence interval; CLD: chronic liver disease; HCC: hepatocellular carcinoma; MALO: major adverse liver outcomes; MASLD: metabolic dysfunction-associated steatotic liver disease PBC: primary biliary cholangitis; PSC: primary sclerosing cholangitis | | | | |
| ^a^ Model 2: conditioned on the matching variables (birth year, sex, county of residence, and calendar year of index date) and further adjusted for country of birth, educational attainment, number of healthcare visits between two years and six months before the index date, and the history of autoimmune diseases. | | | | |

| **Table S7 Subgroup analyses of incident CLD and MALO in patients with CeD and their matched reference individuals** | | | | | |
| --- | --- | --- | --- | --- | --- |
| **Subgroup** | **No. of events**  **(IR, per 100,000 person-years)** | | | **IR difference (95%CI), per 100,000 person-years** | **HR (95%CI) ^a^** |
|  | **Patients** | | **References** |  |  |
|  | Primary outcome | | | | |
|  | Any CLD | | | | |
| Age at index date |  | |  |  |  |
| <18 | 167 (44·7) | | 432 (23·5) | 21·2 (14·1 to 28·3) | 1·95 (1·59 to 2·38) |
| 18-<40 | 182 (102·9) | | 415 (48·8) | 54·1 (38·4 to 69·8) | 2·07 (1·71 to 2·52) |
| 40-<60 | 207 (122·8) | | 508 (62·3) | 60·4 (42·9 to 78·0) | 1·88 (1·58 to 2·24) |
| ≥60 | 93 (95·0) | | 216 (45·3) | 49·7 (29·4 to 69·9) | 2·13 (1·65 to 2·76) |
| Sex |  | |  |  |  |
| Female | 385 (74·1) | | 876 (34·6) | 39·5 (31·8 to 47·3) | 2·14 (1·88 to 2·43) |
| Male | 264 (88·8) | | 695 (48·1) | 40·7 (29·4 to 52·0) | 1·85 (1·59 to 2·17) |
| Born in Nordic countries ^b^ | |  | |  |  |
| Yes | 621 (78·6) | | 1497 (38·9) | 39·7 (33·2 to 46·2) | 2·15 (1·94 to 2·39) |
| No | 28 (103·0) | | 74 (54·9) | 48·1 (8·0 to 88·3) | 1·04 (0·54 to 1·97) |
| Calendar period at index date | |  | |  |  |
| 1969-1989 | 118 (96·4) | | 271 (44·6) | 51·8 (33·6 to 70·0) | 1·99 (1·56 to 2·52) |
| 1990-2001 | 257 (70·4) | | 723 (40·4) | 30·0 (20·9 to 39·1) | 1·66 (1·43 to 1·93) |
| 2002-2009 | 185 (79·8) | | 417 (37·4) | 42·4 (30·4 to 54·5) | 2·04 (1·71 to 2·45) |
| 2010-2017 | 89 (91·1) | | 160 (34·3) | 56·8 (37·2 to 76·5) | 2·73 (2·09 to 3·58) |
| Educational attainment, years | |  | |  |  |
| 0-9 | 107 (102·1) | | 225 (43·7) | 58·4 (38·2 to 78·5) | 1·98 (1·44 to 2·72) |
| 10-12 | 280 (80·9) | | 644 (38·7) | 42·2 (32·3 to 52·2) | 1·72 (1·45 to 2·03) |
| ≥13 | 179 (58·4) | | 535 (36·0) | 22·4 (13·3 to 31·5) | 2·61 (2·07 to 3·28) |
| Missing | 83 (139·3) | | 167 (53·0) | 86·2 (55·2 to 117·3) | 2·59 (1·87 to 3·60) |
| History of autoimmune diseases ^c^ | |  | |  |  |
| Yes | 154 (208·4) | | 171 (45·8) | 162·6 (129·0 to 196·2) | 4·30 (1·89 to 9·77) |
| No | 495 (66·6) | | 1400 (38·8) | 27·8 (21·6 to 34·0) | 1·88 (1·69 to 2·10) |
| History of metabolic-related diseases ^c,d^ | | | |  |  |
| Yes | 101 (156·2) | | 135 (40·5) | 115·7 (84·5 to 146·9) | 3·81 (2·78 to 5·21) |
| No | 548 (72·8) | | 1436 (39·4) | 33·5 (27·0 to 39·9) | 1·88 (1·70 to 2·09) |
|  | Secondary outcomes: specific CLD and MALO | | | | |
|  | Viral hepatitis | | | | |
| Age at index date |  | |  |  |  |
| <18 | 51 (13·6) | | 288 (15·7) | -2·1 (-6·2 to 2·1) | 0·81 (0·56 to 1·15) |
| 18-<40 | 45 (25·3) | | 239 (28·1) | -2·8 (-11·0 to 5·5) | 0·90 (0·64 to 1·27) |
| 40-<60 | 28 (16·5) | | 160 (19·6) | -3·1 (-9·9 to 3·7) | 0·85 (0·56 to 1·29) |
| ≥60 | 10 (10·2) | | 44 (9·2) | 0·9 (-5·9 to 7·8) | 0·89 (0·42 to 1·92) |
| Sex |  | |  |  |  |
| Female | 74 (14·2) | | 419 (16·5) | -2·3 (-5·9 to 1·3) | 0·81 (0·61 to 1·06) |
| Male | 60 (20·1) | | 312 (21·6) | -1·5 (-7·1 to 4·2) | 1·01 (0·75 to 1·37) |
| Born in Nordic countries ^b^ | |  | |  |  |
| Yes | 124 (15·6) | | 685 (17·8) | -2·2 (-5·2 to 0·9) | 0·97 (0·78 to 1·21) |
| No | 10 (36·6) | | 46 (34·1) | 2·6 (-22·2 to 27·3) | 0·43 (0·18 to 1·01) |
| Calendar period at index date | |  | |  |  |
| 1969-1989 | 36 (29·2) | | 114 (18·7) | 10·5 (0·4 to 20·7) | 1·33 (0·83 to 2·14) |
| 1990-2001 | 63 (17·2) | | 365 (20·4) | -3·2 (-7·9 to 1·6) | 0·83 (0·62 to 1·09) |
| 2002-2009 | 27 (11·6) | | 177 (15·9) | -4·3 (-9·2 to 0·7) | 0·84 (0·56 to 1·28) |
| 2010-2017 | 8 (8·2) | | 75 (16·0) | -7·9 (-14·6 to -1·2) | 0·63 (0·30 to 1·32) |
| Educational attainment, years | |  | |  |  |
| 0-9 | 25 (23·8) | | 79 (15·3) | 8·4 (-1·5 to 18·3) | 1·14 (0·62 to 2·08) |
| 10-12 | 61 (17·6) | | 327 (19·6) | -2·1 (-7·0 to 2·8) | 0·75 (0·54 to 1·03) |
| ≥13 | 26 (8·5) | | 292 (19·6) | -11·2 (-15·1 to -7·2) | 0·93 (0·58 to 1·49) |
| Missing | 22 (36·6) | | 33 (10·4) | 26·1 (10·4 to 41·8) | 2·19 (1·01 to 4·74) |
| History of autoimmune diseases ^c^ | |  | |  |  |
| Yes | 30 (40·1) | | 73 (19·5) | 20·6 (5·6 to 35·6) | 1·82 (0·42 to 7·80) |
| No | 104 (13·9) | | 658 (18·2) | -4·3 (-7·3 to -1·3) | 0·83 (0·66 to 1·03) |
| History of metabolic-related diseases ^c,d^ | | | |  |  |
| Yes | 13 (20·0) | | 40 (12·0) | 8·0 (-3·5 to 19·5) | 1·34 (0·54 to 3·36) |
| No | 121 (16·0) | | 691 (18·9) | -2·9 (-6·1 to 0·3) | 0·87 (0·70 to 1·07) |
|  | MASLD | | | | |
| Age at index date |  | |  |  |  |
| <18 | 24 (6·6) | | 64 (3·6) | 3·0 (0·2 to 5·8) | 1·73 (1·04 to 2·88) |
| 18-<40 | 40 (23·2) | | 67 (8·1) | 15·1 (7·7 to 22·6) | 3·20 (2·06 to 4·97) |
| 40-<60 | 42 (25·4) | | 89 (11·2) | 14·3 (6·2 to 22·3) | 2·24 (1·52 to 3·31) |
| ≥60 | 25 (25·9) | | 34 (7·2) | 18·7 (8·2 to 29·1) | 3·27 (1·86 to 5·74) |
| Sex |  | |  |  |  |
| Female | 81 (15·9) | | 158 (6·4) | 9·6 (6·0 to 13·2) | 2·57 (1·92 to 3·44) |
| Male | 50 (17·3) | | 96 (6·8) | 10·5 (5·5 to 15·4) | 2·56 (1·78 to 3·69) |
| Born in Nordic countries ^b^ | |  | |  |  |
| Yes | 122 (15·8) | | 247 (6·6) | 9·2 (6·3 to 12·2) | 2·54 (2·00 to 3·22) |
| No | 9 (34·0) | | 7 (5·3) | 28·7 (6·1 to 51·2) | 2·43 (0·50 to 11·88) |
| Calendar period at index date | |  | |  |  |
| 1969-1989 | 13 (10·9) | | 37 (6·2) | 4·7 (-1·5 to 10·9) | 1·81 (0·88 to 3·69) |
| 1990-2001 | 41 (11·5) | | 98 (5·6) | 5·9 (2·2 to 9·6) | 2·12 (1·42 to 3·16) |
| 2002-2009 | 46 (20·3) | | 85 (7·8) | 12·5 (6·4 to 18·6) | 2·27 (1·55 to 3·31) |
| 2010-2017 | 31 (32·2) | | 34 (7·4) | 24·8 (13·2 to 36·4) | 4·52 (2·72 to 7·52) |
| Educational attainment, years | |  | |  |  |
| 0-9 | 19 (18·7) | | 35 (6·9) | 11·7 (3·0 to 20·4) | 1·82 (0·85 to 3·89) |
| 10-12 | 68 (20·2) | | 111 (6·8) | 13·4 (8·4 to 18·3) | 2·17 (1·52 to 3·09) |
| ≥13 | 35 (11·6) | | 82 (5·6) | 6·0 (1·9 to 10·0) | 2·86 (1·66 to 4·92) |
| Missing | 9 (15·4) | | 26 (8·4) | 7·0 (-3·6 to 17·6) | 2·76 (0·89 to 8·51) |
| History of autoimmune diseases ^c^ | |  | |  |  |
| Yes | 26 (36·4) | | 38 (10·4) | 26·0 (11·6 to 40·4) | 4·55 (0·61 to 34·05) |
| No | 105 (14·4) | | 216 (6·1) | 8·3 (5·5 to 11·2) | 2·63 (2·05 to 3·36) |
| History of metabolic-related diseases ^c,d^ | | | |  |  |
| Yes | 35 (56·1) | | 40 (12·2) | 43·8 (24·9 to 62·8) | 5·33 (2·99 to 9·51) |
| No | 96 (13·0) | | 214 (6·0) | 7·1 (4·3 to 9·8) | 2·26 (1·75 to 2·91) |
|  | Alcohol-related liver disease | | | | |
| Age at index date |  | |  |  |  |
| <18 | 8 (2·1) | | 16 (0·9) | 1·3 (-0·3 to 2·8) | 2·43 (1·00 to 5·90) |
| 18-<40 | 24 (13·5) | | 78 (9·1) | 4·3 (-1·4 to 10·1) | 2·45 (1·28 to 4·69) |
| 40-<60 | 55 (32·3) | | 188 (23·0) | 9·4 (0·2 to 18·5) | 1·36 (0·98 to 1·89) |
| ≥60 | 22 (22·4) | | 81 (17·0) | 5·4 (-4·7 to 15·5) | 1·43 (0·87 to 2·37) |
| Sex |  | |  |  |  |
| Female | 47 (9·0) | | 139 (5·5) | 3·5 (0·8 to 6·3) | 1·65 (1·15 to 2·37) |
| Male | 62 (20·7) | | 224 (15·5) | 5·3 (-0·3 to 10·8) | 1·44 (1·07 to 1·95) |
| Born in Nordic countries ^b^ | |  | |  |  |
| Yes | 106 (13·4) | | 348 (9·0) | 4·3 (1·6 to 7·0) | 1·53 (1·21 to 1·93) |
| No | 3 (11·0) | | 15 (11·1) | -0·1 (-13·7 to 13·5) | NA |
| Calendar period at index date | |  | |  |  |
| 1969-1989 | 20 (16·2) | | 68 (11·2) | 5·1 (-2·5 to 12·6) | 1·47 (0·86 to 2·52) |
| 1990-2001 | 57 (15·5) | | 176 (9·8) | 5·7 (1·4 to 10·0) | 1·47 (1·07 to 2·03) |
| 2002-2009 | 27 (11·6) | | 93 (8·3) | 3·3 (-1·4 to 8·0) | 1·36 (0·88 to 2·11) |
| 2010-2017 | 5 (5·1) | | 26 (5·6) | -0·5 (-5·4 to 4·5) | 0·94 (0·36 to 2·47) |
| Educational attainment, years | |  | |  |  |
| 0-9 | 26 (24·7) | | 70 (13·6) | 11·1 (1·1 to 21·1) | 1·73 (0·96 to 3·11) |
| 10-12 | 37 (10·6) | | 133 (8·0) | 2·7 (-1·0 to 6·4) | 0·99 (0·65 to 1·49) |
| ≥13 | 26 (8·5) | | 93 (6·2) | 2·2 (-1·3 to 5·7) | 2·06 (1·12 to 3·78) |
| Missing | 20 (33·1) | | 67 (21·2) | 12·0 (-3·4 to 27·3) | 1·76 (0·93 to 3·33) |
| History of autoimmune diseases ^c^ | |  | |  |  |
| Yes | 17 (22·7) | | 40 (10·7) | 12·0 (0·7 to 23·2) | NA |
| No | 92 (12·3) | | 323 (8·9) | 3·4 (0·7 to 6·1) | 1·49 (1·16 to 1·90) |
| History of metabolic-related diseases ^c,d^ | | | |  |  |
| Yes | 22 (33·8) | | 37 (11·1) | 22·7 (8·1 to 37·2) | 2·97 (1·63 to 5·42) |
| No | 87 (11·5) | | 326 (8·9) | 2·6 (-0·02 to 5·2) | 1·34 (1·04 to 1·73) |
|  | Autoimmune liver disease | | | | |
| Age at index date |  | |  |  |  |
| <18 | 89 (23·8) | | 73 (4·0) | 19·8 (14·8 to 24·8) | 5·87 (4·18 to 8·25) |
| 18-<40 | 81 (45·6) | | 45 (5·3) | 40·3 (30·3 to 50·4) | 8·24 (5·38 to 12·6) |
| 40-<60 | 82 (48·3) | | 88 (10·8) | 37·6 (26·9 to 48·3) | 4·29 (3·08 to 5·97) |
| ≥60 | 42 (42·8) | | 65 (13·6) | 29·2 (15·8 to 42·5) | 3·24 (2·15 to 4·90) |
| Sex |  | |  |  |  |
| Female | 192 (36·8) | | 183 (7·2) | 29·6 (24·3 to 34·9) | 5·06 (4·07 to 6·29) |
| Male | 102 (34·2) | | 88 (6·1) | 28·1 (21·4 to 34·9) | 4·31 (3·11 to 5·98) |
| Born in Nordic countries ^b^ | |  | |  |  |
| Yes | 288 (36·4) | | 260 (6·8) | 29·6 (25·3 to 33·9) | 5·14 (4·26 to 6·20) |
| No | 6 (21·9) | | 11 (8·1) | 13·8 (-0·4 to 32·0) | NA |
| Calendar period at index date | |  | |  |  |
| 1969-1989 | 57 (46·3) | | 66 (10·8) | 35·5 (23·2 to 47·8) | 3·72 (2·54 to 5·45) |
| 1990-2001 | 102 (27·8) | | 111 (6·2) | 21·7 (16·1 to 27·2) | 4·00 (3·01 to 5·31) |
| 2002-2009 | 88 (37·9) | | 66 (5·9) | 32·0 (23·9 to 40·0) | 5·93 (4·19 to 8·39) |
| 2010-2017 | 47 (48·0) | | 28 (6·0) | 42·0 (28·1 to 55·9) | 6·98 (4·28 to 11·38) |
| Educational attainment, years | |  | |  |  |
| 0-9 | 33 (31·3) | | 48 (9·3) | 22·0 (11·0 to 33·0) | 2·84 (1·50 to 5·37) |
| 10-12 | 127 (36·6) | | 95 (5·7) | 30·9 (24·4 to 37·4) | 5·89 (4·10 to 8·46) |
| ≥13 | 93 (30·3) | | 77 (5·2) | 25·1 (18·8 to 31·4) | 5·81 (3·77 to 8·96) |
| Missing | 41 (68·3) | | 51 (16·1) | 52·2 (30·8 to 73·5) | 3·62 (2·24 to 5·85) |
| History of autoimmune diseases ^c^ | |  | |  |  |
| Yes | 89 (119·6) | | 29 (7·8) | 111·8 (86·8 to 136·8) | 5·09 (1·59 to 16·25) |
| No | 205 (27·5) | | 242 (6·7) | 20·8 (17·0 to 24·7) | 4·57 (3·74 to 5·79) |
| History of metabolic-related diseases ^c,d^ | | | |  |  |
| Yes | 35 (53·8) | | 22 (6·6) | 47·2 (29·2 to 65·2) | 7·72 (4·05 to 14·69) |
| No | 259 (34·3) | | 249 (6·8) | 27·5 (23·2 to 31·8) | 4·80 (3·97 to 5·79) |
|  | MALO | | | | |
| Age at index date |  | |  |  |  |
| <18 | 39 (10·4) | | 104 (5·7) | 4·8 (1·3 to 8·2) | 2·10 (1·41 to 3·13) |
| 18-<40 | 99 (55·6) | | 229 (26·8) | 28·8 (17·3 to 40·3) | 2·16 (1·58 to 2·95) |
| 40-<60 | 217 (127·9) | | 733 (89·7) | 38·2 (20·0 to 56·4) | 1·43 (1·21 to 1·69) |
| ≥60 | 225 (229·6) | | 729 (153·0) | 76·7 (44·7 to 108·7) | 1·62 (1·38 to 1·90) |
| Sex |  | |  |  |  |
| Female | 289 (55·4) | | 991 (39·0) | 16·3 (9·5 to 23·2) | 1·41 (1·22 to 1·61) |
| Male | 291 (97·5) | | 804 (55·5) | 42·0 (30·2 to 53·8) | 1·71 (1·48 to 1·97) |
| Born in Nordic countries ^b^ | |  | |  |  |
| Yes | 548 (69·1) | | 1719 (44·6) | 24·5 (18·3 to 30·6) | 1·53 (1·38 to 1·69) |
| No | 32 (117·2) | | 76 (56·2) | 61·0 (18·5 to 103·5) | 2·43 (1·05 to 5·63) |
| Calendar period at index date | |  | |  |  |
| 1969-1989 | 102 (82·8) | | 273 (44·8) | 38·1 (21·1 to 55·0) | 1·68 (1·31 to 2·16) |
| 1990-2001 | 241 (65·8) | | 848 (47·3) | 18·5 (9·6 to 27·4) | 1·36 (1·17 to 1·57) |
| 2002-2009 | 156 (67·0) | | 486 (43·5) | 23·5 (12·3 to 34·7) | 1·44 (1·19 to 1·73) |
| 2010-2017 | 81 (82·6) | | 188 (40·2) | 42·4 (23·5 to 61·3) | 1·87 (1·43 to 2·44) |
| Educational attainment, years | |  | |  |  |
| 0-9 | 138 (131·2) | | 493 (95·7) | 35·6 (12·1 to 59·0) | 1·40 (1·12 to 1·75) |
| 10-12 | 217 (62·5) | | 585 (35·1) | 27·4 (18·6 to 36·2) | 1·55 (1·29 to 1·87) |
| ≥13 | 112 (36·4) | | 431 (28·9) | 7·5 (0·2 to 14·7) | 1·33 (1·01 to 1·75) |
| Missing | 113 (188·3) | | 286 (90·6) | 97·7 (61·5 to 134·0) | 2·33 (1·79 to 3·04) |
| History of autoimmune diseases ^c^ | |  | |  |  |
| Yes | 132 (176·7) | | 203 (54·3) | 122·4 (91·4 to 153·5) | 3·19 (1·61 to 6·30) |
| No | 448 (60·1) | | 1592 (44·1) | 16·0 (10·1 to 22·0) | 1·45 (1·30 to 1·62) |
| History of metabolic-related diseases ^c,d^ | | | |  |  |
| Yes | 136 (209·4) | | 274 (82·1) | 127·3 (90·8 to 163·8) | 2·43 (1·94 to 3·05) |
| No | 444 (58·8) | | 1521 (41·6) | 17·2 (11·3 to 23·0) | 1·39 (1·24 to 1·56) |
| CeD: celiac disease; CI: confidence interval; CLD: chronic liver disease; HR: hazard ratio; IR: incidence rate; MALO: major adverse liver outcomes; MASLD: metabolic dysfunction-associated steatotic liver disease  ^a^ Model 2: conditioned on the matching variables (birth year, sex, county of residence, and calendar year) and further adjusted for country of birth, educational attainment, number of healthcare visits between two years and six months before the index date, and history of autoimmune diseases.  ^b^ Nordic countries: Sweden, Denmark, Finland, Norway, and Iceland.  ^c^ Codes for metabolic-related diseases and autoimmune diseases are listed in Table S4. ^d^ Metabolic-related diseases: hypertension, diabetes, obesity, and dyslipidemia. | | | | | |

**Table S8 Any or specific CLD before incident MALO while following patients with CeD, n (%)**

|  | **CeD patients who developed any MALO** | **CeD patients who did not develop any MALO** |
| --- | --- | --- |
| Primary outcome |  |  |
| Any CLD | 138 (23·8%) | 470 (1·0%) |
| Secondary outcomes: specific CLD |  |  |
| Viral hepatitis | 13 (2·2%) | 119 (0·3%) |
| MASLD | 19 (3·3%) | 104 (0·2%) |
| Alcohol-related liver disease | 50 (8·6%) | 32 (0·1%) |
| Autoimmune liver disease | 62 (10·7%) | 216 (0·5%) |
| AIH | 27 (4·7%) | 87 (0·2%) |
| PBC | 17 (2·9%) | 33 (0·1%) |
| PSC | 22 (3·8%) | 112 (0·2%) |
| AIH: autoimmune hepatitis; CeD: celiac disease; CLD: chronic liver disease; MALO: major adverse liver outcomes; MASLD: metabolic dysfunction-associated steatotic liver disease; PBC: primary biliary cholangitis; PSC: primary sclerosing cholangitis. | | |

**Table S9 Incident CLD and MALO in childhood-onset patients diagnosed with CeD before 2012**

|  | **No. of events (IR, per 100,000 person-years)** | | **IR difference (95%CI), per 100,000 person-years** | **HR (95%CI)** | |
| --- | --- | --- | --- | --- | --- |
|  | **Patients** | **References** |  | **Model 1 ^a^** | **Model 2 ^b^** |
| Primary outcome |  |  |  |  |  |
| Any CLD | 155 (43·9) | 415 (23·9) | 20·0 (12·7 to 27·3) | 1·99 (1·64 to 2·42) | 1·79 (1·46 to 2·20) |
| Secondary outcomes: specific CLD and MALO | |  |  |  |  |
| Viral hepatitis | 50 (14·1) | 278 (16·0) | -1·9 (-6·2 to 2·5) | 0·90 (0·65 to 1·25) | 0·82 (0·58 to 1·16) |
| MASLD | 21 (6·1) | 60 (3·5) | 2·6 (-0·2 to 5·3) | 1·89 (1·14 to 3·14) | 1·59 (0·92 to 2·72) |
| Alcohol-related liver disease | 7 (2·0) | 16 (0·9) | 1·1 (-0·5 to 2·6) | 2·16 (0·89 to 5·25) | 2·05 (0·81 to 5·20) |
| Autoimmune liver disease | 82 (23·2) | 71 (4·1) | 19·1 (14·0 to 24·2) | 6·02 (4·34 to 8·35) | 5·37 (3·80 to 7·57) |
| AIH | 38 (10·7) | 33 (1·9) | 8·8 (5·4 to 12·3) | 5·81 (3·60 to 9·38) | 5·38 (3·27 to 8·85) |
| PBC | 5 (1·4) | 3 (0·2) | 1·2 (0·01 to 2·5) | NA | NA |
| PSC | 48 (13·6) | 42 (2·4) | 11·1 (7·2 to 15·1) | 6·07 (3·97 to 9·29) | 5·49 (3·52 to 8·58) |
| MALO | 37 (10·4) | 99 (5·7) | 4·8 (1·2 to 8·3) | 2·06 (1·39 to 3·05) | 2·10 (1·40 to 3·14) |
| Cirrhosis, compensated or decompensated | 31 (8·8) | 88 (5·1) | 3·7 (0·4 to 7·0) | 1·97 (1·29 to 3·00) | 2·02 (1·30 to 3·12) |
| HCC | 5 (1·4) | 7 (0·4) | 1·0 (-0·3 to 2·3) | 2·29 (0·60 to 8·79) | 1·77 (0·43 to 7·26) |
| Liver transplantation | 6 (1·7) | 9 (0·5) | 1·2 (-0·2 to 2·6) | 3·33 (1·12 to 9·92) | 3·52 (1·17 to 10·56) |
| Liver-related death | 2 (0·6) | 3 (0·2) | 0·4 (-0·4 to 1·2) | NA | NA |
| AIH: autoimmune hepatitis; CeD: celiac disease; CI: confidence interval; CLD: chronic liver disease; HCC: hepatocellular carcinoma; HR: hazard ratio; IR: incidence rate; MALO: major adverse liver outcomes; MASLD: metabolic dysfunction-associated steatotic liver disease; PBC: primary biliary cholangitis; PSC: primary sclerosing cholangitis | | | | | |
| ^a^ Model 1: conditioned on the matching variables (birth year, sex, county of residence, and calendar year of index date). | | | | | |
| ^b^ Model 2: model 1+further adjusted for country of birth, educational attainment, number of healthcare visits between two years and six months before the index date, and history of autoimmune diseases. | | | | | |

**Table S10 Sensitivity analyses of incident CLD and MALO in patients** with CeD and their matched reference individuals

|  | **Only individuals with available educational attainment** | | |  | **Excluding first one year of follow-up** | | | **Excluding first three years of follow-up** | | | | | **Lifetime risk of CLD or MALO ^b^** | | | | |
| --- | --- | --- | --- | --- | --- | --- | --- | --- | --- | --- | --- | --- | --- | --- | --- | --- | --- |
|  | **No. of events (IR, per 100,000 person-years)** | | **HR (95%CI) ^a^** |  | **No. of events (IR, per 100,000 person-years)** | | **HR (95%CI) ^a^** | |  | **No. of events (IR, per 100,000 person-years)** | | **HR (95%CI) ^a^** | | **With/without CLD or MALO** | | | **OR (95%CI) ^c^** |
|  | **Patients** | **References** |  |  | **Patients** | **References** |  |  |  | **Patients** | **References** |  |  |  | **Patients** | **References** |  |
| Primary outcome |  |  |  |  |  |  |  | |  |  |  |  | |  |  |  |  |
| Any CLD | 566 (74·7) | 1369 (37·4) | 1·99 (1·79 to 2·21) |  | 592 (72·5) | 1495 (37·6) | 1·90 (1·72 to 2·11) | |  | 505 (62·0) | 1364 (34·4) | 1·75 (1·56 to 1·96) | |  | 1266/48560 | 2968/242662 | 1·77 (1·62 to 1·95) |
| Secondary outcomes: specific CLD and MALO | | | |  |  |  |  | |  |  |  |  | |  |  |  |  |
| Viral hepatitis | 112 (14·7) | 665 (18·1) | 0·83 (0·67 to 1·03) |  | 122 (14·9) | 683 (17·2) | 0·85 (0·68 to 1·05) | |  | 103 (12·6) | 606 (15·3) | 0·75 (0·58 to 0·96) | |  | 334/49492 | 1566/244064 | 0·86 (0·72 to 1·02) |
| MASLD | 122 (16·5) | 226 (6·3) | 2·57 (2·04 to 3·24) |  | 121 (15·2) | 245 (6·3) | 2·44 (1·93 to 3·09) | |  | 105 (13·2) | 234 (6·0) | 2·19 (1·71 to 2·81) | |  | 178/49648 | 310/245320 | 2·48 (2·01 to 3·06) |
| Alcohol-related liver disease | 89 (11·7) | 296 (8·1) | 1·47 (1·15 to 1·89) |  | 103 (12·5) | 351 (8·8) | 1·48 (1·17 to 1·88) | |  | 88 (10·7) | 331 (8·3) | 1·29 (0·99 to 1·67) | |  | 241/49585 | 701/244929 | 1·25 (1·04 to 1·52) |
| Autoimmune liver disease | 253 (33·3) | 219 (6·0) | 5·15 (4·24 to 6·26) |  | 263 (32·1) | 261 (6·5) | 4·38 (3·63 to 5·28) | |  | 221 (27·0) | 233 (5·9) | 4·07 (3·31 to 5·01) | |  | 625/49201 | 551/245079 | 4·59 (3·88 to 5·44) |
| AIH | 102 (13·4) | 95 (2·6) | 5·02 (3·72 to 6·78) |  | 111 (13·5) | 108 (2·7) | 4·72 (3·53 to 6·32) | |  | 93 (11·4) | 97 (2·4) | 4·26 (3·09 to 5·86) | |  | 329/49497 | 318/245312 | 5·63 (4·36 to 7·26) |
| PBC | 45 (5·9) | 75 (2·0) | 3·04 (2·07 to 4·47) |  | 47 (5·7) | 91 (2·3) | 2·49 (1·70 to 3·64) | |  | 37 (4·5) | 79 (2·0) | 2·00 (1·28 to 3·14) | |  | 142/49684 | 144/245486 | 3·32 (2·43 to 4·53) |
| PSC | 125 (16·4) | 66 (1·8) | 7·71 (5·53 to 10·75) |  | 129 (15·7) | 81 (2·0) | 1·56 (1·19 to 2·05) | |  | 113 (13·8) | 75 (1·9) | 5·85 (4·19 to 8·17) | |  | 239/49587 | 132/245498 | 5·50 (4·08 to 7·41) |
| MALO | 467 (61·4) | 1501 (40·9) | 1·46 (1·31 to 1·62) |  | 522 (63·7) | 1735 (43·5) | 1·40 (1·26 to 1·55) | |  | 460 (56·2) | 1598 (40·2) | 1·32 (1·18 to 1·47) | |  | 1056/48770 | 2554/243076 | 1·46 (1·33 to 1·60) |
| Cirrhosis, compensated or decompensated | 413 (54·3) | 1294 (35·3) | 1·50 (1·34 to 1·69) |  | 455 (55·5) | 1493 (37·5) | 1·43 (1·28 to 1·60) | |  | 404 (49·4) | 1380 (34·7) | 1·37 (1·21 to 1·54) | |  | 945/48881 | 2233/243397 | 1·50 (1·36 to 1·65) |
| HCC | 44 (5·8) | 147 (4·0) | 1·40 (0·98 to 1·98) |  | 53 (6·5) | 180 (4·5) | 1·32 (0·95 to 1·84) | |  | 46 (5·6) | 164 (4·1) | 1·19 (0·82 to 1·71) | |  | 110/49716 | 260/245370 | 1·54 (1·19 to 1·99) |
| Liver transplantation | 22 (2·9) | 33 (0·9) | 2·73 (1,51 to 4·94) |  | 25 (3·0) | 35 (0·9) | 2·71 (1·53 to 4·81) | |  | 23 (2·8) | 32 (0·8) | 2·97 (1·62 to 5·42) | |  | 92/49734 | 46/245584 | 5·88 (3·76 to 9·19) |
| Liver-related death | 86 (11·3) | 323 (8·8) | 1·29 (1·01 to 1·65) |  | 114 (13·9) | 403 (10·1) | 1·34 (1·07 to 1·67) | |  | 100 (12·2) | 371 (9·3) | 1·37 (1·21 to 1·54) | |  | 229/49597 | 586/245044 | 1·70 (1·44 to 2·00) |
| AIH: autoimmune hepatitis; CeD: celiac disease; CI: confidence interval; CLD: chronic liver disease; HCC: hepatocellular carcinoma; HR: hazard ratio; IR: incidence rate; MALO: major adverse liver outcomes; MASLD: metabolic dysfunction-associated steatotic liver disease; OR: odds ratio; PBC: primary biliary cholangitis; PSC: primary sclerosing cholangitis  ^a^ Model 2: conditioned on matching variables (birth year, sex, county of residence, and calendar year) and further adjusted for country of birth, educational attainment, number of healthcare visits between two years and six months before the index date, and history of autoimmune diseases.  ^b^ CeD was treated as a time-invariant exposure.  ^c^ Conditioned on covariates in model 2 and additionally adjusted for the temporal relationship between CeD and CLD/MALO. | | | | | | | | | | | | | | | | | |

**Table S11** Characteristics of patients with CeD and their siblings, n (%)

|  | **Patients with CeD** | **Full siblings** |
| --- | --- | --- |
| N | 31176 | 52999 |
| Age at index date, years |  |  |
| Mean ± SD | 26·4 ± 20·9 | 29·7 ± 20·7 |
| Median (IQR) | 21·3 (7·8-43·2) | 27·0 (10·7-47·1) |
| <18 | 14128 (45·3%) | 20056 (37·8%) |
| 18-<40 | 8135 (26·1%) | 14734 (27·8%) |
| 40-<60 | 6383 (20·5%) | 13271 (25·0%) |
| ≥60 | 2530 (8·1%) | 4938 (9·3%) |
| Female | 19809 (63·5%) | 26065 (49·2%) |
| Born in Nordic countries ^a^ |  |  |
| Yes | 30705 (98·5%) | 51703 (97·6%) |
| No | 471 (1·5%) | 1290 (2·4%) |
| Missing | 0 | 6 (0·0%) |
| Calendar period at index date |  |  |
| 1969-1989 | 2198 (7·1%) | 3985 (7·5%) |
| 1990-2001 | 9902 (31·8%) | 17632 (33·3%) |
| 2002-2009 | 10798 (34·6%) | 18137 (34·2%) |
| 2010-2017 | 8278 (26·6%) | 13245 (25·0%) |
| Educational attainment, years |  |  |
| 0-9 | 3086 (9·9%) | 6956 (13·1%) |
| 10-12 | 13097 (42·0%) | 22489 (42·4%) |
| ≥13 | 13953 (44·8%) | 20720 (39·1%) |
| Missing | 1040 (3·3%) | 2834 (5·3%) |
| Number of healthcare visits ^b^ |  |  |
| 0 | 17816 (57·1%) | 36586 (69·0%) |
| 1 | 5216 (16·7%) | 7322 (13·8%) |
| 2-3 | 4170 (13·4%) | 5035 (9·5%) |
| ≥4 | 3974 (12·7%) | 4056 (7·7%) |
| History of metabolic-related diseases ^c^ | 3137 (10·1%) | 3720 (7·0%) |
| Hypertension | 1403 (4·5%) | 2490 (4·7%) |
| Diabetes | 1673 (5·4%) | 1111 (2·1%) |
| Obesity | 178 (0·6%) | 355 (0·7%) |
| Dyslipidemia | 827 (2·7%) | 1450 (2·7%) |
| History of autoimmune diseases ^c^ | 3398 (10·9%) | 2169 (4·1%) |
| Follow-up time, years |  |  |
| Mean ± SD | 17·4 ± 8·4 | 17·4 ± 8·7 |
| Median (IQR) | 16·3 (10·8-23·1) | 16·4 (10·6-23·3) |
| 0-0·9 | 193 (0·6%) | 715 (1·3%) |
| 1-4·9 | 654 (2·1%) | 1566 (3·0%) |
| 5-9·9 | 5821 (18·7%) | 9694 (18·3%) |
| 10-19·9 | 13583 (43·6%) | 22105 (41·7%) |
| ≥20 | 10925 (35·0%) | 18919 (35·7%) |
| CeD: celiac disease; IQR: interquartile range: SD: standard deviation.  ^a^ Nordic countries: Sweden, Denmark, Finland, Norway, and Iceland.  ^b^ Between two years and six months before the index date.  ^c^ Codes for metabolic-related diseases and autoimmune diseases are listed in Table S4. | | |

| **Table S12 Incident CLD and MALO in patients with CeD and their full siblings** | | | | | |
| --- | --- | --- | --- | --- | --- |
|  | **No. of events (IR, per 100,000 person-years)** | | **IR difference (95%CI), per 100,000 person-years** | **HR (95%CI)** | |
|  | **Patients** | **Full siblings** |  | **Model 1 ^a^** | **Model 2 ^b^** |
| Primary outcome |  |  |  |  |  |
| Any CLD | 427 (78·6) | 403 (43·8) | 34·8 (26·2 to 43·4) | 2·20 (1·90 to 2·55) | 1·96 (1·68 to 2·27) |
| Secondary outcomes: specific CLD and MALO | | |  |  |  |
| Viral hepatitis | 85 (15·6) | 146 (15·9) | -0·3 (-4·5 to 3·9) | 1·09 (0·81 to 1·46) | 0.99 (0·73 to 1·35) |
| MASLD | 88 (16·6) | 68 (7·6) | 9·0 (5·1 to 12·9) | 2·63 (1·88 to 3·68) | 2·41 (1·71 to 3·39) |
| Alcohol-related liver disease | 68 (12·4) | 93 (10·1) | 2·4 (-1·2 to 6·0) | 1·87 (1·32 to 2·63) | 1.73 (1·22 to 2·46) |
| Autoimmune liver disease | 199 (36·5) | 102 (11·1) | 25·4 (19·9 to 30·9) | 3·72 (2·89 to 4·78) | 3.07 (2·37 to 3·97) |
| AIH | 82 (15·0) | 45 (4·9) | 10·1 (6·6 to 13·7) | 3·42 (2·32 to 5·02) | 3.16 (2·14 to 4·67) |
| PBC | 38 (7·0) | 22 (2·4) | 4·6 (2·1 to 7·0) | 3·51 (1·95 to 6·34) | 3.41 (1·87 to 6·20) |
| PSC | 95 (17·4) | 43 (4·7) | 12·7 (9·0 to 16·5) | 4·26 (2·93 to 6·20) | 2.93 (1·98 to 4·32) |
| MALO | 302 (55·3) | 412 (44·7) | 10·6 (3·0 to 18·2) | 1·60 (1·37 to 1·87) | 1·43 (1·22 to 1·68) |
| Cirrhosis, compensated or decompensated | 272 (49·8) | 379 (41·1) | 8·7 (1·4 to 15·9) | 1·56 (1·33 to 1·84) | 1·42 (1·20 to 1·67) |
| HCC | 30 (5·5) | 39 (4·2) | 1,3 (-1·1 to 3·6) | 1·69 (1·02 to 2·80) | 1·47 (0·87 to 2·47) |
| Liver transplantation | 19 (3·5) | 12 (1·3) | 2·2 (0·4 to 3·9) | 3·07 (1·39 to 6·81) | 2·60 (1·15 to 5·88) |
| Liver-related death | 47 (8·6) | 90 (9·8) | -1,2 (-4·3 to 2·0) | 1·09 (0·73 to 1·62) | 0·93 (0·62 to 1·39) |
| AIH: autoimmune hepatitis; CeD: celiac disease; CI: confidence interval; CLD: chronic liver disease; HCC: hepatocellular carcinoma; HR: hazard ratio; IR: incidence rate; MALO: major adverse liver outcomes; MASLD: metabolic dysfunction-associated steatotic liver disease; PBC: primary biliary cholangitis; PSC: primary sclerosing cholangitis | | | | | |
| ^a^ Model 1: conditioned on the matching variables (birth year, sex, county of residence, and calendar year of index date). | | | | | |
| ^b^ Model 2: model 1+further adjusted for country of birth, educational attainment, number of healthcare visits between two years and six months before the index date, and history of autoimmune diseases. | | | | | |

| **Table S13 Subgroup analyses of incident CLD and MALO in patients with CeD and their full siblings** | | | | | |
| --- | --- | --- | --- | --- | --- |
| **Subgroup** | | **No. of events**  **(IR, per 100,000 person-years)** | | **IR difference (95%CI), per 100,000 person-years** | **HR (95%CI) ^a^** |
|  |  | **Patients** | **Full siblings** |  |  |
|  | | Primary outcome | | | |
|  | | Any CLD | | | |
| Age at index date | |  |  |  |  |
| <18 | | 122 (46·8) | 100 (27·3) | 19·5 (9·6 to 29·4) | 1·93 (1·42 to 2·63) |
| 18-<40 | | 138 (99·4) | 113 (45·9) | 53·5 (34·9 to 72·1) | 2·32 (1·75 to 3·08) |
| 40-<60 | | 132 (115·7) | 152 (62·1) | 53·6 (31·5 to 75·7) | 1·84 (1·44 to 2·36) |
| ≥60 | | 35 (117·4) | 38 (60·3) | 57·1 (13·7 to 100·5) | 2·15 (1·29 to 3·60) |
| Sex | |  |  |  |  |
| Female | | 250 (72·1) | 251 (43·0) | 29·1 (18·7 to 39·5) | 2·22 (1·74 to 2·83) |
| Male | | 177 (90·0) | 152 (45·3) | 44·8 (29·7 to 59·8) | 1·82 (1·39 to 2·40) |
| Born in Nordic countries ^b^ |  | | |  |  |
| Yes | | 423 (78·8) | 392 (43·4) | 35·4 (26·8 to 44·0) | 1·98 (1·70 to 2·30) |
| No | | 4 (64·1) | 11 (69·8) | -5·8 (-80·9 to 69·4) | 0·70 (0·19 to 2·53) |
| Calendar period at index date |  | | |  |  |
| 1969-1989 | | 69 (95·5) | 60 (46·5) | 49·0 (23·6 to 74·4) | 2·19 (1·50 to 3·20) |
| 1990-2001 | | 170 (72·6) | 175 (43·0) | 29·6 (16·9 to 42·2) | 1·74 (1·39 to 2·17) |
| 2002-2009 | | 130 (78·9) | 125 (46·3) | 32·6 (16·8 to 48·4) | 1·80 (1·39 to 2·34) |
| 2010-2017 | | 58 (80·5) | 43 (37·8) | 42·8 (19·2 to 66·4) | 2·36 (1·56 to 3·55) |
| Educational attainment, years |  | | |  |  |
| 0-9 | | 59 (107·1) | 82 (66·6) | 40·5 (9·6 to 71·4) | 1·38 (0·91 to 2·09) |
| 10-12 | | 196 (83·7) | 190 (48·6) | 35·1 (21·5 to 48·7) | 1·75 (1·38 to 2·21) |
| ≥13 | | 130 (57·9) | 94 (27·9) | 30·0 (18·6 to 41·5) | 2·63 (1·90 to 3·65) |
| Missing | | 42 (141·5) | 37 (53·7) | 87·8 (41·7 to 133·9) | 3·41 (1·70 to 6·82) |
| History of autoimmune diseases ^c^ |  | | |  |  |
| Yes | | 105 (202·5) | 58 (61·9) | 140·6 (98·7 to 182·5) | 1·84 (0·89 to 3·82) |
| No | | 322 (65·5) | 345 (41·8) | 23·8 (15·3 to 32·2) | 2·01 (1·70 to 2·37) |
| History of metabolic-related diseases ^c,d^ | | | |  |  |
| Yes | | 62 (154·7) | 39 (52·6) | 102·1 (60·2 to 144·0) | 3·18 (1·98 to 5·12) |
| No | | 365 (72·5) | 364 (43·0) | 29·5 (20·8 to 38·1) | 1·89 (1·61 to 2·21) |
|  | | Secondary outcomes: specific CLD and MALO | | | |
|  | | Viral hepatitis | | | |
| Age at index date | |  |  |  |  |
| <18 | | 33 (12·6) | 53 (14·5) | -1·9 (-7·7 to 4·0) | 0·93 (0·55 to 1·60) |
| 18-<40 | | 34 (24·4) | 49 (19·9) | 4·5 (-5·4 to 14·4) | 1·34 (0·81 to 2·20) |
| 40-<60 | | 16 (13·9) | 40 (16·3) | -2·4 (-10·9 to 6·1) | 0·86 (0·47 to 1·58) |
| ≥60 | | 2 (6·7) | 4 (6·3) | 0·3 (-10·8 to 11·5) | NA |
| Sex | |  |  |  |  |
| Female | | 47 (13·5) | 94 (16·1) | -2·6 (-7·6 to 2·5) | 1·13 (0·69 to 1·86) |
| Male | | 38 (19·2) | 52 (15·5) | 3·8 (-3·7 to 11·2) | 1·27 (0·75 to 2·16) |
| Born in Nordic countries ^b^ |  | | |  |  |
| Yes | | 82 (15·2) | 140 (15·5) | -0·3 (-4·4 to 3·9) | 0·97 (0·71 to 1·32) |
| No | | 3 (48·1) | 6 (38·0) | 10·0 (-52·3 to 72·3) | 1·09 (0·24 to 5·01) |
| Calendar period at index date |  | | |  |  |
| 1969-1989 | | 23 (31·6) | 28 (21·6) | 10·0 (-5·2 to 25·2) | 1·21 (0·64 to 2·28) |
| 1990-2001 | | 40 (17·0) | 69 (16·9) | 0·07 (-6·5 to 6·7) | 0·94 (0·62 to 1·43) |
| 2002-2009 | | 16 (9·7) | 39 (14·4) | -4·8 (-11·3 to 1·8) | 0·76 (0·41 to 1·40) |
| 2010-2017 | | 6 (8·3) | 10 (8·8) | -0·5 (-9·1 to 8·1) | NA |
| Educational attainment, years |  | | |  |  |
| 0-9 | | 16 (28·9) | 29 (23·5) | 5·4 (-11·1 to 22·0) | 1·18 (0·56 to 2·49) |
| 10-12 | | 37 (15·7) | 72 (18·4) | -2·7 (-9·3 to 3·9) | 0·78 (0·49 to 1·25) |
| ≥13 | | 19 (8·4) | 33 (9·8) | -1·4 (-6·4 to 3·7) | 1·16 (0·61 to 2·20) |
| Missing | | 13 (43·3) | 12 (17·3) | 25·9 (0·4 to 51·4) | 2·61 (0·53 to 12·83) |
| History of autoimmune diseases ^c^ |  | | |  |  |
| Yes | | 20 (38·1) | 25 (26·6) | 11·5 (-8·2 to 31·2) | 2·07 (0·27 to 15·96) |
| No | | 65 (13·2) | 121 (14·6) | -1·5 (-5·6 to 2·7) | 1·07 (0·77 to 1·50) |
| History of metabolic-related diseases ^c,d^ | | | |  |  |
| Yes | | 6 (14·9) | 14 (18·9) | -4·0 (-19·5 to 11·4) | NA |
| No | | 79 (15·6) | 132 (15·6) | 0·04 (-4·3 to 4·4) | 1·02 (0·75 to 1·40) |
|  | | MASLD | | | |
| Age at index date | |  |  |  |  |
| <18 | | 19 (7·5) | 18 (5·0) | 2·4 (-1·6 to 6·5) | 2·53 (0·88 to 7·30) |
| 18-<40 | | 30 (22·1) | 16 (6·6) | 15·5 (6·9 to 24·0) | 4·40 (2·07 to 9·35) |
| 40-<60 | | 27 (24·1) | 27 (11·2) | 12·9 (2·8 to 22·9) | 2·16 (1·21 to 3·85) |
| ≥60 | | 12 (41·0) | 7 (11·3) | 29·7 (5·0 to 54·3) | 3·99 (1·40 to 11·35) |
| Sex | |  |  |  |  |
| Female | | 54 (15·9) | 45 (7·9) | 8·0 (3·2 to 12·9) | 2·08 (1·26 to 3·45) |
| Male | | 34 (17·7) | 23 (7·0) | 10·7 (4·1 to 17·3) | 3·20 (1·52 to 6·74) |
| Born in Nordic countries ^b^ |  | | |  |  |
| Yes | | 88 (16·8) | 66 (7·5) | 9·3 (5·4 to 13·2) | 2·47 (1·74 to 3·49) |
| No | | 0 (0) | 2 (13·1) | -13·1 (-31·1 to 5·1) | NA |
| Calendar period at index date |  | | |  |  |
| 1969-1989 | | 6 (8·5) | 9 (7·1) | 1·4 (-6·8 to 9·7) | 1·14 (0·26 to 5·05) |
| 1990-2001 | | 27 (11·8) | 28 (7·0) | 4·8 (-0·4 to 10·0) | 1·73 (0·99 to 3·05) |
| 2002-2009 | | 35 (21·7) | 17 (6·4) | 15·3 (7·4 to 23·1) | 5·13 (2·22 to 11·84) |
| 2010-2017 | | 20 (28·1) | 14 (12·4) | 15·7 (1·8 to 29·7) | 2·48 (1·21 to 5·06) |
| Educational attainment, years |  | | |  |  |
| 0-9 | | 12 (22·5) | 15 (12·6) | 9·9 (-4·3 to 24·1) | 1·81 (0·66 to 4·97) |
| 10-12 | | 45 (19·7) | 29 (7·6) | 12·1 (5·7 to 18·5) | 2·83 (1·52 to 5·27) |
| ≥13 | | 27 (12·2) | 19 (5·7) | 6·5 (1·2 to 11·8) | 2·63 (1·26 to 5·52) |
| Missing | | 4 (13·8) | 5 (7·4) | 6·4 (-8·6 to 21·3) | NA |
| History of autoimmune diseases ^c^ |  | | |  |  |
| Yes | | 18 (36·0) | 4 (4·4) | 31·6 (14·4 to 48·8) | NA |
| No | | 70 (14·6) | 64 (7·9) | 6·6 (2·7 to 10·6) | 2·18 (1·50 to 3·15) |
| History of metabolic-related diseases ^c,d^ | | | |  |  |
| Yes | | 26 (67·6) | 4 (5·5) | 62·0 (35·5 to 88·5) | 14·68 (4·47 to 48·22) |
| No | | 62 (12·6) | 64 (7·7) | 4·9 (1·2 to 8·5) | 2·03 (1·36 to 3·04) |
|  | | Alcohol-related liver disease | | | |
| Age at index date | |  |  |  |  |
| <18 | | 4 (1·5) | 4 (1·1) | 0·4 (-1·4 to 2·3) | NA |
| 18-<40 | | 18 (12·9) | 21 (8·5) | 4·4 (-2·6 to 11·3) | 2·84 (1·23 to 6·59) |
| 40-<60 | | 36 (31·3) | 52 (21·2) | 10·1 (-1·6 to 21·9) | 1·93 (1·19 to 3·12) |
| ≥60 | | 10 (33·4) | 16 (25·3) | 8·0 (-16·1 to 32·1) | 1·46 (0·62 to 3·43) |
| Sex | |  |  |  |  |
| Female | | 29 (8·3) | 57 (9·7) | -1·4 (-5·4 to 2·5) | 2·38 (1·23 to 4·58) |
| Male | | 39 (19·7) | 36 (10·7) | 9·0 (1·9 to 16·1) | 1·64 (0·94 to 2·87) |
| Born in Nordic countries ^b^ |  | | |  |  |
| Yes | | 67 (12·4) | 93 (10·3) | 2·1 (-1·5 to 5·8) | 1·68 (1·18 to 2·39) |
| No | | 1 (15·9) | 0 (0) | 15·9 (-15·3 to 47·1) | NA |
| Calendar period at index date |  | | |  |  |
| 1969-1989 | | 10 (13·7) | 16 (12·3) | 1·4 (-9·1 to 11·8) | 1·00 (0·40 to 2·53) |
| 1990-2001 | | 38 (16·1) | 40 (9·8) | 6·3 (0·4 to 12·3) | 2·19 (1·35 to 3·55) |
| 2002-2009 | | 18 (10·9) | 28 (10·4) | 0·5 (-5·8 to 6·8) | 1·55 (0·82 to 2·91) |
| 2010-2017 | | 2 (2·8) | 9 (7·9) | -5·1 (-11·6 to 1·3) | NA |
| Educational attainment, years |  | | |  |  |
| 0-9 | | 12 (21·6) | 23 (18·6) | 3·0 (-11·4 to 17·4) | 0·91 (0·40 to 2·04) |
| 10-12 | | 26 (11·0) | 43 (11·0) | 0·1 (-5·3 to 5·4) | 1·81 (0·97 to 3·39) |
| ≥13 | | 21 (9·3) | 13 (3·9) | 5·5 (1·0 to 10·0) | 7·13 (2·24 to 22·7) |
| Missing | | 9 (29·8) | 14 (20·2) | 9·7 (-12·5 to 31·8) | 0·43 (0·07 to 2·58) |
| History of autoimmune diseases ^c^ |  | | |  |  |
| Yes | | 15 (28·5) | 16 (17·0) | 11·5 (-5·2 to 28·1) | 0·79 (0·21 to 2·92) |
| No | | 53 (10·7) | 77 (9·3) | 1·4 (-2·1 to 5·0) | 1·78 (1·20 to 2·63) |
| History of metabolic-related diseases ^c,d^ | | | |  |  |
| Yes | | 15 (37·2) | 13 (17·5) | 19·6 (-1·4 to 40·7) | 2·33 (0·99 to 5·47) |
| No | | 53 (10·5) | 80 (9·4) | 1·0 (-2·5 to 4·5) | 1·87 (1·24 to 2·81) |
|  | | Autoimmune liver disease | | | |
| Age at index date | |  |  |  |  |
| <18 | | 70 (26·8) | 25 (6·8) | 20·0 (13·2 to 26·8) | 4·12 (2·50 to 6·80) |
| 18-<40 | | 64 (45·9) | 31 (12·6) | 33·3 (21·2 to 45·4) | 3·21 (1·99 to 5·17) |
| 40-<60 | | 52 (45·3) | 32 (13·1) | 32·3 (19·2 to 45·4) | 3·03 (1·89 to 4·85) |
| ≥60 | | 13 (43·4) | 14 (22·2) | 21·3 (-5·1 to 47·6) | 2·16 (0·86 to 5·42) |
| Sex | |  |  |  |  |
| Female | | 128 (36·8) | 63 (10·8) | 26·0 (19·1 to 32·9) | 3·29 (2·20 to 4·92) |
| Male | | 71 (36·0) | 39 (11·6) | 24·4 (15·3 to 33·5) | 2·60 (1·54 to 4·38) |
| Born in Nordic countries ^b^ |  | | |  |  |
| Yes | | 199 (36·9) | 99 (10·9) | 26·0 (20·4 to 31·6) | 3·16 (2·43 to 4·10) |
| No | | 0 (0) | 3 (18·9) | -18·9 (-40·4 to 24·9) | NA |
| Calendar period at index date |  | | |  |  |
| 1969-1989 | | 35 (48·2) | 11 (8·5) | 39·7 (23·0 to 56·5) | 5·95 (2·91 to 12·16) |
| 1990-2001 | | 68 (28·9) | 39 (9·6) | 19·4 (11·9 to 26·8) | 3·20 (2·08 to 4·93) |
| 2002-2009 | | 64 (38·7) | 40 (14·8) | 23·9 (13·4 to 34·5) | 2·26 (1·48 to 3·46) |
| 2010-2017 | | 32 (44·4) | 12 (10·5) | 33·8 (17·3 to 50·3) | 4·11 (2·06 to 8·18) |
| Educational attainment, years |  | | |  |  |
| 0-9 | | 17 (30·7) | 16 (13·0) | 17·7 (1·8 to 33·7) | 2·03 (0·79 to 5·20) |
| 10-12 | | 97 (41·3) | 51 (13·0) | 28·3 (19·3 to 37·2) | 2·64 (1·78 to 3·91) |
| ≥13 | | 64 (28·4) | 28 (8·3) | 20·1 (12·5 to 27·8) | 3·43 (2·00 to 5·89) |
| Missing | | 21 (70·2) | 7 (10·1) | 60·1 (29·2 to 91·1) | 6·73 (2·38 to 19·01) |
| History of autoimmune diseases ^c^ |  | | |  |  |
| Yes | | 61 (116·8) | 12 (12·8) | 104·1 (73·9 to 134·3) | 4·28 (1·13 to 16·16) |
| No | | 138 (28·0) | 90 (10·9) | 17·1 (11·9 to 22·3) | 3·23 (2·39 to 4·36) |
| History of metabolic-related diseases ^c,d^ | | | |  |  |
| Yes | | 18 (44·6) | 8 (10·8) | 33·8 (11·9 to 55·8) | 3·39 (1·13 to 10·15) |
| No | | 181 (35·9) | 94 (11·1) | 24·8 (19·1 to 30·4) | 3·17 (2·43 to 4·14) |
|  | | MALO | | | |
| Age at index date | |  |  |  |  |
| <18 | | 26 (9·9) | 23 (6·3) | 3·7 (-0·9 to 8·3) | 2·02 (1·04 to 3·95) |
| 18-<40 | | 71 (50·8) | 67 (27·1) | 23·7 (10·2 to 37·2) | 1·93 (1·26 to 2·95) |
| 40-<60 | | 143 (124·6) | 225 (91·8) | 32·8 (9·1 to 56·5) | 1·40 (1·11 to 1·76) |
| ≥60 | | 62 (207·5) | 97 (153·9) | 53·6 (-6·5 to 113·6) | 1·24 (0·88 to 1·73) |
| Sex | |  |  |  |  |
| Female | | 147 (42·2) | 246 (42·0) | 0·2 (-8·5 to 8·8) | 1·38 (1·06 to 1·80) |
| Male | | 155 (78·5) | 166 (49·4) | 29·1 (14·7 to 43·6) | 1·66 (1·25 to 2·19) |
| Born in Nordic countries ^b^ |  | | |  |  |
| Yes | | 298 (55·2) | 408 (45·0) | 10·2 (2·5 to 17·8) | 1·41 (1·20 to 1·66) |
| No | | 4 (63·8) | 4 (25·3) | 38·5 (-28·7 to 105·7) | NA |
| Calendar period at index date |  | | |  |  |
| 1969-1989 | | 43 (59·1) | 47 (36·3) | 22·8 (2·3 to 43·3) | 2·11 (1·24 to 3·59) |
| 1990-2001 | | 126 (53·5) | 174 (42·7) | 10·9 (-0·4 to 22·1) | 1·36 (1·07 to 1·73) |
| 2002-2009 | | 84 (50·8) | 146 (54·0) | -3·3 (-17·2 to 10·7) | 1·14 (0·86 to 1·51) |
| 2010-2017 | | 49 (67·8) | 45 (39·5) | 28·4 (6·1 to 50·6) | 1·94 (1·27 to 2·96) |
| Educational attainment, years |  | | |  |  |
| 0-9 | | 57 (103·0) | 99 (80·2) | 22·8 (-8·3 to 53·9) | 0·99 (0·68 to 1·45) |
| 10-12 | | 131 (55·7) | 183 (46·7) | 9·0 (-2·7 to 20·7) | 1·53 (1·16 to 2·02) |
| ≥13 | | 70 (31·1) | 86 (25·5) | 5·6 (-3·5 to 14·6) | 1·64 (1·09 to 2·48) |
| Missing | | 44 (146·9) | 44 (63·6) | 83·2 (35·9 to 130·5) | 8·65 (1·90 to 39·3) |
| History of autoimmune diseases ^c^ |  | | |  |  |
| Yes | | 84 (160·3) | 64 (68·2) | 92·2 (54·0 to 130·3) | 1·16 (0·62 to 2·16) |
| No | | 218 (44·2) | 348 (42·0) | 2·1 (-5·2 to 9·5) | 1·38 (1·15 to 1·66) |
| History of metabolic-related diseases ^c, d^ | | | |  |  |
| Yes | | 71 (176·5) | 53 (71·5) | 105·0 (59·7 to 150·3) | 2·42 (1·63 to 3·60) |
| No | | 231 (45·7) | 359 (42·4) | 3·3 (-4·0 to 10·7) | 1·26 (1·05 to 1·52) |
| CeD: celiac disease; CI: confidence interval; CLD: chronic liver disease; HR: hazard ratio; IR: incidence rate; MALO: major adverse liver outcomes; MASLD: metabolic dysfunction-associated steatotic liver disease.  ^a^ Model 2: conditioned on the matching variables (birth year, sex, county of residence, and calendar year) and further adjusted for country of birth, educational attainment, number of healthcare visits between two years and six months before the index date, and history of autoimmune diseases.  ^b^ Nordic countries: Sweden, Denmark, Finland, Norway, and Iceland.  ^c^ Codes for metabolic-related diseases and autoimmune diseases are listed in Table S4. ^d^ Metabolic-related diseases: hypertension, diabetes, obesity, and dyslipidemia. | | | | | |

**Table S14 Characteristics of CeD patients who had a follow-up biopsy within six months** and five years after diagnosis, n (%)

|  | **Persistent villus atrophy** | **Mucosal healing** |
| --- | --- | --- |
| N | 2768 | 6624 |
| Age at index date, years |  |  |
| Mean ± SD | 43·7 ± 24·3 | 32·3 ± 22·5 |
| Median (IQR) | 47·5 (23·7-63·9) | 30·0 (11·8-50·3) |
| <18 | 557 (20·1%) | 2127 (32·1%) |
| 18-<40 | 540 (19·5%) | 2055 (31·0%) |
| 40-<60 | 816 (29·5%) | 1488 (22·5%) |
| ≥60 | 855 (30·9%) | 954 (14·4%) |
| Female | 1651 (59·6%) | 4324 (65·3%) |
| Born in Nordic countries ^a^ |  |  |
| Yes | 2640 (95·4%) | 6332 (95·6%) |
| No | 128 (4·6%) | 292 (4.4%) |
| Missing | 0 | 0 |
| Calendar period at the date of follow-up biopsy |  |  |
| 1969-1989 | 168 (6·1%) | 234 (3·5%) |
| 1990-2001 | 1026 (37·1%) | 1994 (30·1%) |
| 2002-2009 | 902 (32·6%) | 2503 (37·8%) |
| 2010-2017 | 672 (24·3%) | 1893 (28·6%) |
| Educational attainment, years |  |  |
| 0-9 | 587 (21·2%) | 728 (11·0%) |
| 10-12 | 1159 (41·9%) | 2812 (42·5%) |
| ≥13 | 907 (32·8%) | 2936 (44·3%) |
| Missing | 115 (4·2%) | 148 (2·2%) |
| Number of healthcare visits ^b^ |  |  |
| 0 | 864 (31·2%) | 1727 (26·1%) |
| 1 | 537 (19·4%) | 1285 (19·4%) |
| 2-3 | 611 (22·1%) | 1545 (23·3%) |
| ≥4 | 756 (27·3%) | 2067 (31·2%) |
| History of metabolic-related diseases ^c^ | 414 (15·0%) | 780 (11·8%) |
| Hypertension | 287 (10·4%) | 460 (6·9%) |
| Diabetes | 123 (4·4%) | 295 (4·5%) |
| Obesity | 13 (0·5%) | 51 (0·8%) |
| Dyslipidemia | 169 (6·1%) | 307 (4·6%) |
| History of autoimmune diseases ^c^ | 389 (14·1%) | 849 (12·8%) |
| Follow-up time, years |  |  |
| Mean ± SD | 16·5 ± 8·6 | 16·0 ± 7·7 |
| Median (IQR) | 15·6 (9·5-22·6) | 15·3 (9·8-21·0) |
| 0-0·9 | 34 (1·2%) | 44 (0·7%) |
| 1-4·9 | 128 (4·6%) | 198 (3·0%) |
| 5-9·9 | 590 (21·3%) | 1469 (22·2%) |
| 10-19·9 | 1112 (40·2%) | 3023 (45·6%) |
| ≥20 | 904 (32·7%) | 1890 (28·5%) |
| CeD: celiac disease; IQR: interquartile range: SD: standard deviation.  ^a^ Nordic countries: Sweden, Denmark, Finland, Norway, and Iceland.  ^b^ Within two years and six months before the date of follow-up biopsy.  ^c^ Codes for metabolic-related diseases and autoimmune diseases are listed in Table S4. | | |

| **Table S15 Incident CLD and MALO in CeD patients who had a follow-up biopsy after diagnosis** | | | | | |
| --- | --- | --- | --- | --- | --- |
|  | **No. of events (IR, per 100,000 person-years)** | | **IR difference (95%CI), per 100,000 person-years** | **HR (95%CI)** | |
|  | **Persistent villus atrophy** | **Mucosal healing** |  | **Model 1 ^a^** | **Model 2 ^b^** |
| Primary outcome |  |  |  |  |  |
| Any CLD | 48 (105·3) | 89 (84·0) | 21·3 (-13·3 to 55·8) | 1·17 (0·80 to 1·70) | 1·13 (0·77 to 1·64) |
| Secondary outcomes: specific CLD and MALO | | | |  |  |
| Viral hepatitis | 9 (19·7) | 13 (12·2) | 7·4 (-7·0 to 21·9) | 1·83 (0·73 to 4·60) | 1·63 (0·65 to 4·09) |
| MASLD | 8 (17·9) | 24 (23·2) | -5·2 (-20·7 to 10·3) | 0·72 (0·32 to 1·67) | 0·72 (0·31 to 1·64) |
| Alcohol-related liver disease | 10 (21·8) | 15 (14·1) | 7·7 (-7·6 to 23·0) | 1·12 (0·48 to 2·62) | 1·09 (0·47 to 2·55) |
| Autoimmune liver disease | 21 (45·8) | 38 (35·7) | 10·1 (-12·6 to 32·8) | 1·15 (0·64 to 2·07) | 1·13 (0·63 to 2·03) |
| AIH | 9 (19·6) | 15 (14·1) | 5·5 (-9·1 to 20·2) | 0·63 (0·14 to 2·94) | 0·64 (0·14 to 2·96) |
| PBC | 3 (6·5) | 5 (4·7) | 1·8 (-6·6 to 10·3) | NA | NA |
| PSC | 9 (19·6) | 22 (20·7) | -1·0 (-16·5 to 14·4) | 0·95 (0·41 to 2·20) | 0·92 (0·39 to 2·12) |
| MALO | 52 (113·5) | 63 (59·2) | 54·4 (20·2 to 88·5) | 1·29 (0·88 to 1·89) | 1·27 (0·87 to 1·87) |
| Cirrhosis, compensated or decompensated | 42 (91·7) | 56 (52·6) | 39·1 (8·2 to 70·1) | 1·18 (0·78 to 1·79) | 1·19 (0·78 to 1·80) |
| HCC | 9 (19·6) | 5 (4·7) | 14·9 (1·5 to 28·4) | 2·38 (0·74 to 7·67) | 1·99 (0·62 to 6·46) |
| Liver transplantation | 2 (4·4) | 4 (3·8) | 0·6 (-6·5 to 7·7) | NA | NA |
| Liver-related death | 16 (34·8) | 7 (6·6) | 28·3 (10·5 to 46·0) | 2·68 (1·02 to 7·01) | 2·74 (1·04 to 7·20) |
| AIH: autoimmune hepatitis; CeD: celiac disease; CI: confidence interval; CLD: chronic liver disease; HCC: hepatocellular carcinoma; HR: hazard ratio; IR: incidence rate; MALO: major adverse liver outcomes; MASLD: metabolic dysfunction-associated steatotic liver disease; PBC: primary biliary cholangitis; PSC: primary sclerosing cholangitis | | | | | |
| ^a^ Model 1: conditioned on the matching variables (birth year, sex, county of residence, and calendar year of index date) and the duration between CeD diagnosis and the follow-up biopsy. | | | | | |
| ^b^ Model 2: model 1+further adjusted for country of birth, educational attainment, number of healthcare visits between two years and six months before the index date, history of autoimmune diseases, and the duration between CeD diagnosis and follow-up biopsy. | | | | | |

**References**

1. Aggarwal N, Agarwal A, Alarouri H, Dwarakanathan V, Dang S, Ahuja V, et al. Patients with Celiac Disease Have High Prevalence of Fatty Liver and Metabolic Syndrome. Digestive Diseases and Sciences. 2024.

2. Jena A, Kumar MP, Kumar A, Birda CL, Choudhury A, Kumar N, et al. Liver abnormalities in celiac disease and response to gluten free diet: A systematic review and meta-analysis. J Gastroenterol Hepatol. 2023;38(1):11-22.

3. Habash N, Choung RS, Jacobson RM, Murray JA, Absah I. Celiac Disease: Risk of Hepatitis B Infection. J Pediatr Gastroenterol Nutr. 2022;74(3):328-32.

4. Rinella ME, Lazarus JV, Ratziu V, Francque SM, Sanyal AJ, Kanwal F, et al. A multisociety Delphi consensus statement on new fatty liver disease nomenclature. Journal of Hepatology. 2023;79(6):1542-56.

5. Hitawala AA, Almomani A, Onwuzo S, Boustany A, Kumar P, Asaad I. Prevalence of autoimmune, cholestatic and nonalcoholic fatty liver disease in celiac disease. Eur J Gastroenterol Hepatol. 2023;35(9):1030-36.

6. Hitawala A, Onwuzo S, Almomani A, Alchirazi KA, Krishtopaytis E, Boustany A, et al. S1264 Prevalence and Risk Factors Associated With Non-Alcoholic Fatty Liver Disease in Patients With Celiac Disease. The American journal of gastroenterology. 2022;117(10S):e912-e13.

7. Voss J, Schneider CV, Kleinjans M, Bruns T, Trautwein C, Strnad P. Hepatobiliary phenotype of individuals with chronic intestinal disorders. Sci Rep. 2021;11(1):19954.

8. Reilly NR, Lebwohl B, Hultcrantz R, Green PH, Ludvigsson JF. Increased risk of non-alcoholic fatty liver disease after diagnosis of celiac disease. J Hepatol. 2015;62(6):1405-11.

9. Ludvigsson JF, Elfstrom P, Broome U, Ekbom A, Montgomery SM. Celiac disease and risk of liver disease: a general population-based study. Clin Gastroenterol Hepatol. 2007;5(1):63-69 e1.

10. Conrad N, Misra S, Verbakel JY, Verbeke G, Molenberghs G, Taylor PN, et al. Incidence, prevalence, and co-occurrence of autoimmune disorders over time and by age, sex, and socioeconomic status: a population-based cohort study of 22 million individuals in the UK. Lancet. 2023;401(10391):1878-90.

11. Lawson A, West J, Aithal GP, Logan RF. Autoimmune cholestatic liver disease in people with coeliac disease: a population-based study of their association. Aliment Pharmacol Ther. 2005;21(4):401-5.

12. West J, Logan RF, Card TR, Smith C, Hubbard R. Fracture risk in people with celiac disease: a population-based cohort study. Gastroenterology. 2003;125(2):429-36.

13. Sorensen HT, Thulstrup AM, Blomqvist P, Nørgaard B, Fonager K, Ekbom A. Risk of primary biliary liver cirrhosis in patients with coeliac disease: Danish and Swedish cohort data. Gut. 1999;44(5):736-8.

14. Kingham JGC, Parker DR. The association between primary biliary cirrhosis and coeliac disease: a study of relative prevalences. Gut. 1998;42(1):120-22.

15. Lebwohl B, Green PHR, Emilsson L, Mårild K, Söderling J, Roelstraete B, et al. Cancer Risk in 47,241 Individuals With Celiac Disease: A Nationwide Cohort Study. Clin Gastroenterol Hepatol. 2022;20(2):e111-e31.

16. Yoosuf S, Singh P, Khaitan A, Strand TA, Ahuja V, Makharia GK. Prevalence of Celiac Disease in Patients With Liver Diseases: A Systematic Review and Meta-Analyses. Am J Gastroenterol. 2023;118(5):820-32.

17. Haggård L, Glimberg I, Lebwohl B, Sharma R, Verna EC, Green PHR, et al. High prevalence of celiac disease in autoimmune hepatitis: Systematic review and meta-analysis. Liver Int. 2021;41(11):2693-702.

18. Vajro P, Paolella G, Maggiore G, Giordano G. Pediatric celiac disease, cryptogenic hypertransaminasemia, and autoimmune hepatitis. J Pediatr Gastroenterol Nutr. 2013;56(6):663-70.

19. Lundberg Bave A, von Seth E, Ingre M, Nordenvall C, Bergquist A. Autoimmune diseases in primary sclerosing cholangitis and their first-degree relatives. Hepatology. 2024;80(3):527-35.

20. Roderburg C, Loosen S, Kostev K, Demir M, Joerdens MS, Luedde T. Nonalcoholic fatty liver disease is associated with a higher incidence of coeliac disease. Eur J Gastroenterol Hepatol. 2022;34(3):328-31.

21. Renno A, Abdel-Aziz Y, Alastal Y, Khuder S, Hasan S, Assaly R, et al. The association between obstructive sleep apnea and non-alcoholic steatohepatitis: A retrospective nationwide inpatient sample analysis. Clinical and Experimental Hepatology. 2021;7(1):25-29.

22. Grønbæk L, Vilstrup H, Deleuran B, Wiest R, Krag A, Jepsen P. Alcoholic Cirrhosis Increases Risk for Autoimmune Diseases: A Nationwide Registry-Based Cohort Study. Clin Gastroenterol Hepatol. 2015;13(11):2017-22.

23. Ludvigsson JF, Brandt L, Montgomery SM, Granath F, Ekbom A. Validation study of villous atrophy and small intestinal inflammation in Swedish biopsy registers. BMC Gastroenterology. 2009;9(1):19.

24. Lebwohl B, Haggård L, Emilsson L, Söderling J, Roelstraete B, Butwicka A, et al. Psychiatric Disorders in Patients With a Diagnosis of Celiac Disease During Childhood From 1973 to 2016. Clinical Gastroenterology and Hepatology. 2021;19(10):2093-101.e13.

25. Hagstrom H, Vessby J, Ekstedt M, Shang Y. 99% of patients with NAFLD meet MASLD criteria and natural history is therefore identical. J Hepatol. 2024;80(2):e76-e77.

26. Miao J, Brismar K, Nyren O, Ugarph-Morawski A, Ye W. Elevated hip fracture risk in type 1 diabetic patients: a population-based cohort study in Sweden. Diabetes Care. 2005;28(12):2850-5.
